# Decrease of *Pdzrn3* is required for heart maturation and protects against heart failure

**DOI:** 10.1038/s41598-021-03795-7

**Published:** 2022-01-07

**Authors:** Mathieu Pernot, Béatrice Jaspard-vinassa, Alice Abelanet, Sebastien Rubin, Isabelle Forfar, Sylvie Jeanningros, Laura Cetran, Murielle Han-Yee Yu, Elise Balse, Stéphane Hatem, Pascale Dufourcq, Thierry Couffinhal, Cécile Duplàa

**Affiliations:** 1 Univ. Bordeaux, Inserm, UMR1034, Biology of Cardiovascular Diseases, 1 Avenue de Magellan, 33600 Pessac, France; 2 Faculté de Médecine, Université Pierre et Marie Curie, Sorbonne University, INSERM UMR_S1166, Paris, France; 3 Service de Biochimie Clinique, CHU de Bordeaux, Bordeaux, France; 4 Service des Maladies Cardiaques et Vasculaires, CHU de Bordeaux, Bordeaux, France

**Keywords:** Cardiovascular biology, Cardiomyopathies, Cardiac hypertrophy, Disease model, Basolateral polarity, Molecular medicine

## Abstract

Heart failure is the final common stage of most cardiopathies. Cardiomyocytes (CM) connect with others via their extremities by intercalated disk protein complexes. This planar and directional organization of myocytes is crucial for mechanical coupling and anisotropic conduction of the electric signal in the heart. One of the hallmarks of heart failure is alterations in the contact sites between CM. Yet no factor on its own is known to coordinate CM polarized organization. We have previously shown that PDZRN3, an ubiquitine ligase E3 expressed in various tissues including the heart, mediates a branch of the Planar cell polarity (PCP) signaling involved in tissue patterning, instructing cell polarity and cell polar organization within a tissue. PDZRN3 is expressed in the embryonic mouse heart then its expression dropped significantly postnatally corresponding with heart maturation and CM polarized elongation. A moderate CM overexpression of Pdzrn3 (*Pdzrn3* OE) during the first week of life, induced a severe eccentric hypertrophic phenotype with heart failure. In models of pressure-overload stress heart failure, CM-specific *Pdzrn3* knockout showed complete protection against degradation of heart function. We reported that Pdzrn3 signaling induced PKC ζ expression, c-Jun nuclear translocation and a reduced nuclear ß catenin level, consistent markers of the planar non-canonical Wnt signaling in CM. We then show that subcellular localization (intercalated disk) of junction proteins as Cx43, ZO1 and Desmoglein 2 was altered in *Pdzrn3* OE mice, which provides a molecular explanation for impaired CM polarization in these mice. Our results reveal a novel signaling pathway that controls a genetic program essential for heart maturation and maintenance of overall geometry, as well as the contractile function of CM, and implicates PDZRN3 as a potential therapeutic target for the prevention of human heart failure.

## Introduction

Cardiomyocytes (CM) can be seen as highly polarized cells with specialized structures present only between the ends of each abutted cell, the intercalated disks (IDs). These regions provide cell-to-cell mechanical connections and mediate electrochemical communication^1^. During the embryonic stage, myocardial cells start to be locally coordinated and aligned in a planar bias^2^. Initially spherical, CM gradually elongate during fetal and postnatal development, to adopt a rod shape. Most components of ID are initially expressed all around the fetal cardiomyocyte membrane and, after birth, following the elongation process, they progressively reorganize from the lateral cell membrane to the ends of each cell. The remodeling and polarization of these specialized organized regions of the plasma membrane is only achieved after birth.

Planar cell polarity (PCP) signaling is a main pathway involved in tissue patterning, instructing cell polarity and cell polar organization within a tissue^3^. It ensures a coordinated planar polarization between contacting cells. Initially identified in Drosophila, the PCP pathway is well conserved in mammalian species. PCP signaling involves a multiprotein complex that associates at the cell membrane, including Frizzled, Disheveled (Dvl), Vangl (Vangl), and Scribble (Scrib). PCP pathways are important in polarized cell migration and organ morphogenesis through activation of cytoskeletal pathways, such as those involving the small GTPases RhoA and Rac, protein kinase C, and Jun N-terminal kinase.

It is also involved in regulating cardiogenesis in vertebrates^4^. Genetic mouse studies have demonstrated that PCP components such as Wnt11, Vangl2, Scrib, Dvl2 and Rac1 regulate cardiomyocyte polarity and embryonic heart development^5–7^. Deletion of Wnt-11 and Vangl2 reveals its critical role in cell adhesion required for organization of CM in the developing ventricular wall^5,6^.

We have previously shown that PDZRN3, an ubiquitine ligase E3 mediates a branch of the PCP pathway involved in vascular polarization and morphogenesis, and in the stabilization of endothelial cell/cell junctions^8,9^. Results from us and other groups, demonstrated that Pdzrn3 was a direct target of the non-canonical PCP Wnt signaling axis, independent of the β catenin^10^. However, the role of PCP in postnatal myocyte shape maturation and polarized ID reorganization has not yet been investigated. We aimed at a deeper understanding of the mechanisms governing postnatal establishment and maintenance of these IDs, and alterations during the development of heart failure.

In the present study, we demonstrate that PDZRN3 is highly expressed in the embryonic mouse heart, then its expression dropped significantly postnatally corresponding with heart maturation. A moderate overexpression of Pdzrn3 (*Pdzrn3* OE) during the first week of life, induced a severe eccentric hypertrophic phenotype with heart failure. In models of pressure-overload stress heart failure where *Pdzrn3* expression is re-induced, CM-specific *Pdzrn3* knockout showed complete protection against degradation of heart function. We showed that Pdzrn3 is a critical regulator of CM maturation and stabilization of IDs and plays a major role in the transition from concentric to eccentric hypertrophy toward heart failure. Biochemical analysis on heart lysates reveals that PDZRN3 signaling regulates PKCζ expression and c-Jun nuclear translocation, reduces β catenin in the nucleus and impairs cytoskeletal actin remodeling, consistent markers of the non-canonical Wnt signaling in CM. We then show that subcellular localization of cardiomyocyte junction proteins as Cx43, ZO1 and Desmoglein 2 was altered in *Pdzrn3* OE mice, which provides a molecular explanation for impaired cardiomyocyte polarization in these mice. This study identifies PDZRN3 as a novel signaling regulator of polarized myocyte organization and opens this pathway up to the development of novel therapeutic strategies for preventing heart failure pathologies.

## Results

### Myocardial *Pdzrn3* reactivation induces cardiac eccentric hypertrophy

**Figure 1:**
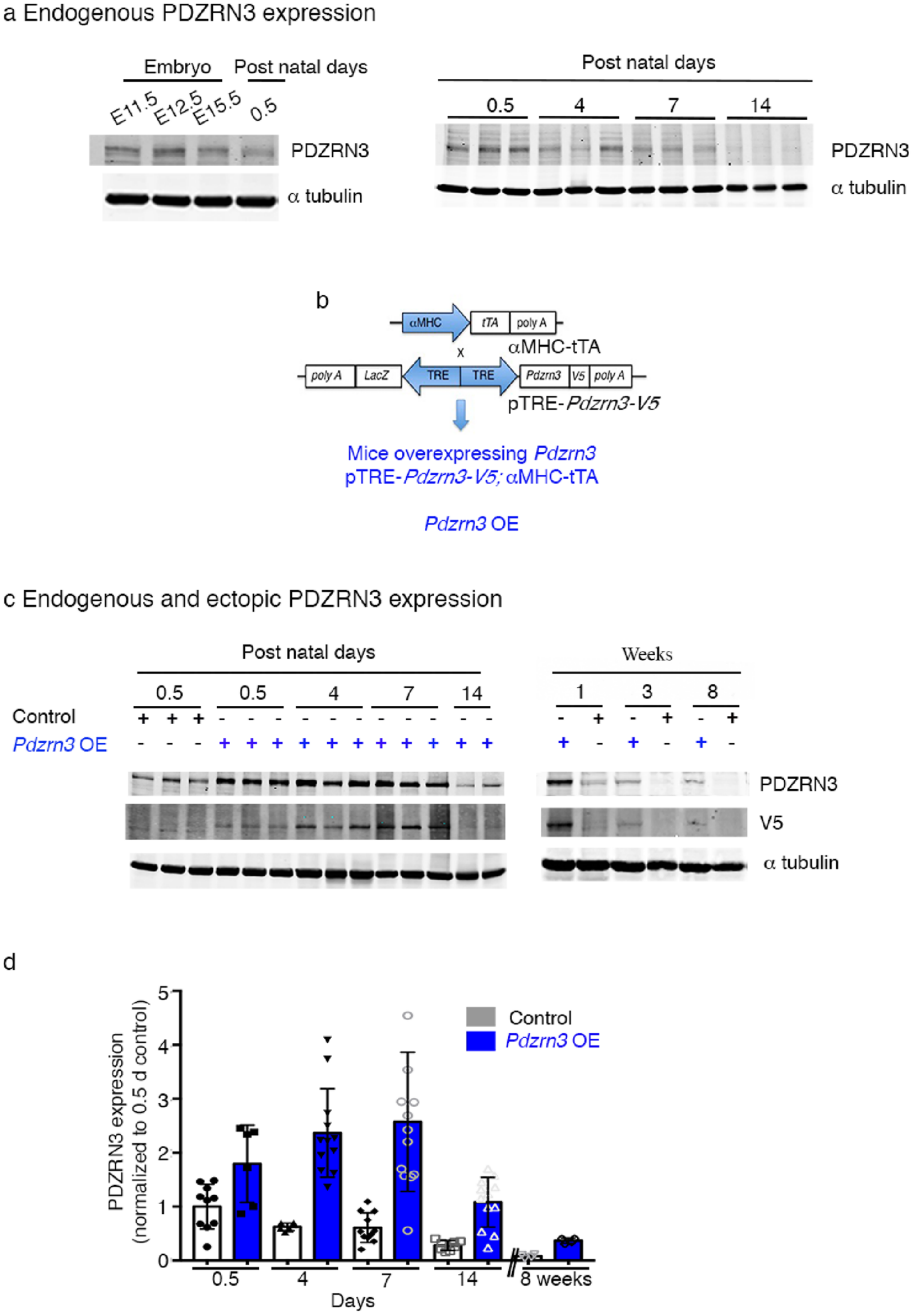
Physiological PDZRN3 expression around birth. Results of P*dzrn3* overexpression. (**a**) Representative Western blot demonstrates levels of PDZRN3 in the embryo at E11.5, 12.5 and 15.5 days; and after birth (0.5, 4, 7 and 14 days). αtubulin was used as the loading control. (**b**) Schematic of *Pdzrn3* overexpression (OE) in the heart. Control mice were (MHC-tTA, *Pdzrn3* OE mice were pTRE-*Pdzrn3*; αMHC-tTA. (**c**) Representative Western blot demonstrates levels of endogenous PDZRN as well as overexpression from 0.5 to 14 days and at 8 weeks after birth. αtubulin was used as the loading control. (**d**) The ratio is quantified and calibrated to the average of 0.5 d control mice. Data are expressed as mean ± s.e.m. (n = 6–13).

*Pdzrn3* was initially identified by two-hybrid screening of a mouse 12.5 embryonic day (E12.5) heart cDNA library^8^. To understand the function of *Pdzrn3* induction in cardiac disease, we analyzed myocardial expression patterns of PDZRN3 during embryogenesis and in postnatal weeks. Western blot analysis confirmed that PDZRN3 is strongly expressed in the embryonic mouse heart (E 11.5 to 15.5), but its expression dropped significantly postnatally from day (d) 0.5 to 14 d, becoming faintly expressed in the adult mouse heart (2 months). This kinetic corresponds with heart maturation (Fig. 1a).

To address its role in cardiac maturation, we employed a gain-of-function approach to overexpress Pdzrn3 in postnatal myocardium. Mice overexpressing Pdzrn3 (*Pdzrn3* OE) were generated by crossing reporter mice, which harbor a bidirectional *Tet*-promoter cassette with genes for PDZRN3-V5 and β galactosidase, with *αMHC-tTA* mice to allow for specific expression of *Pdzrn3* in CM at birth. (Fig. 1b) We used Western blot to examine the expression pattern of ectopic PDZRN3 related to endogenous PDZRN3 levels during postnatal maturation. Ectopic PDZRN3 could be detected after birth at 0.5 d, and peaked between 4 and 7 d by approximately 2- and threefold, respectively. After 14 d, ectopic PDZRN3 decreased to levels seen endogenously at 0.5 days, and maintained low levels of ectopic PDZRN3 expression into adulthood (Fig. 1c,d).

**Figure 2:**
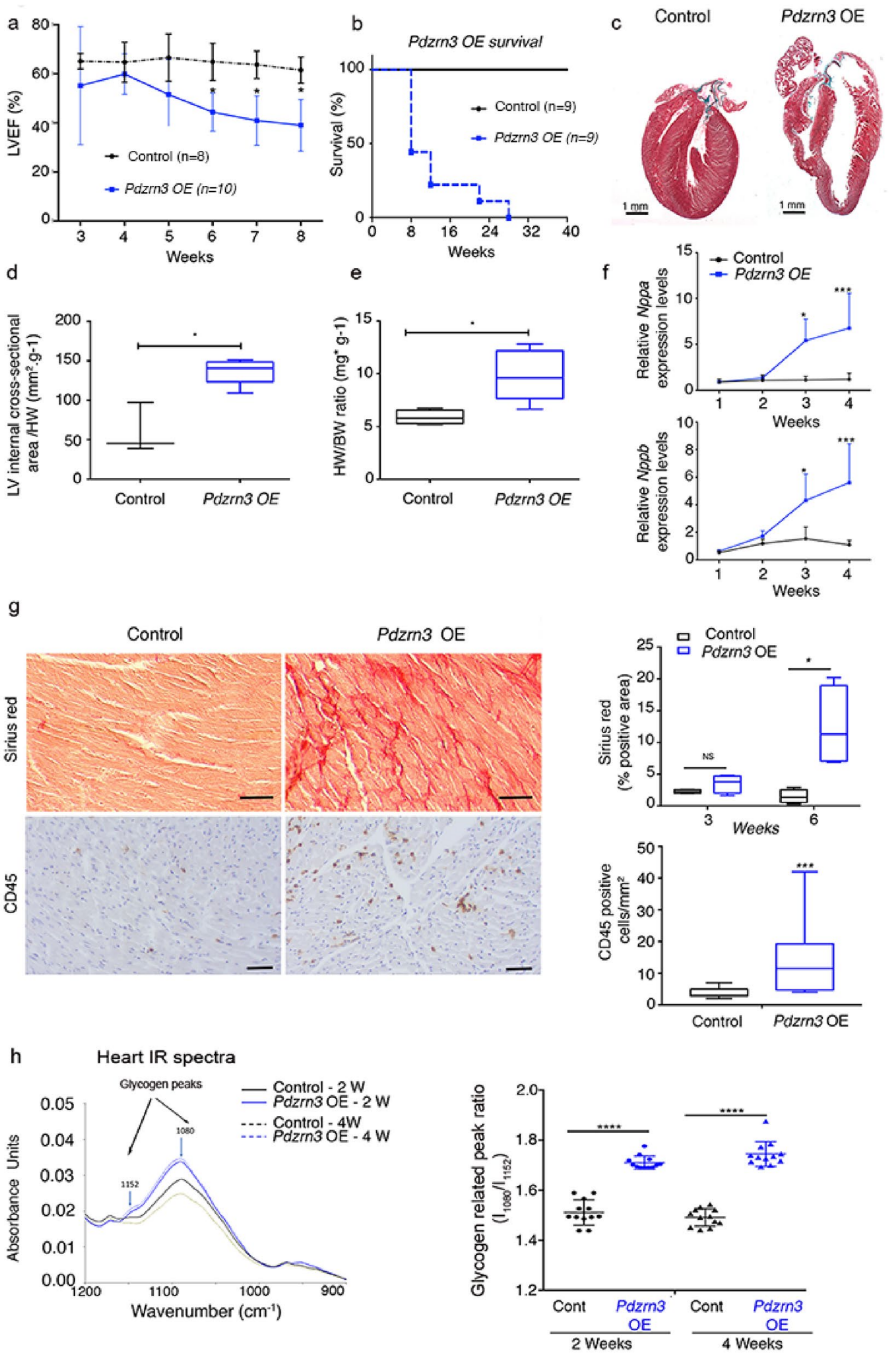
Myocardial reactivation of *Pdzrn3* induces eccentric cardiac hypertrophy. (**a**) LVEF was quantified from 3 to 8 weeks of age (control, n = 4; *Pdzrn3* OE, n = 7). (**b**) Kaplan–Meier curve showing survival rate of control and *Pdzrn3* OE mice from birth until 40 weeks of age (control and *Pdzrn3* OE groups, n = 9). (**c**) Representative images of hematoxylin and eosin (H&E) stained sections of control and *Pdzrn3* OE hearts at 6 weeks, demonstrate that Pdzrn3 OE mice have enlarged hearts. (**d**) Increased LV internal cross section (control, n = 3; *Pdzrn3* OE, n = 5). (**e**) Increased heart weight (HW) to body weight (BW) ratio (control, n = 5; *Pdzrn3* OE, n = 5). (**f**) Increase of the mRNA levels of atrial (Nppa) and brain (Nppb) natriuretic peptide in *Pdzrn3* OE versus control mice over time. mRNA levels were normalized to cyclophiline and are expressed as a relative expression/fold increase over levels found in control mice at 1 week (n = 3–7 mice per group). (**g**) In hearts from *Pdzrn3* OE mice, quantification of total collagen deposition on picrosirius red staining revealing an increase in fibrosis and CD45 immunolabeling, and an increase of inflammatory cells at 6 and 4 weeks of age, respectively (n = 4 mice per group) (the scale bars represent 50 μm). (**h**) FTIR spectra after pre-processing and classification (n = 6 spectra per condition) in the 900 cm^−1^ to 1200 cm^−1^ spectral region obtained heart tissue cryo-sections retrieved from the left ventricle from control and *Pdzrn3* OE mice (n = 3 per group at 2 and 4 weeks). Arrows indicate the wavenumbers studied. Changes in the heart glycogen illustrated with the absorbance ratio 1080 cm^−1^/1152 cm^−1^. All the data are represented as mean ± sem, with n = 3 mice. Mean ± s.e.m. *P < 0.05, **P < 0.005, ***P < 0.001 by repeated-measures two way ANOVA with post-hoc sidak’s test (**a**, **f**); Kaplan–Meier nonparametric regression analysis and the log-rank test (**b**) and unpaired *t*-test (**d**, **e**, **g**).

We observed that LVEF was progressively impaired as early as 4 weeks, in *Pdzrn3* OE mice compared with control mice, resulting in nearly 100% mortality at 28 weeks of age (Fig. 2a,b and Table 1). Surface electrocardiograms (ECGs) performed on 3–10-week-old *Pdzrn3* OE mice and their control littermates showed no differences in heart rate or RR, QRS and QTc intervals. However, *Pdzrn3* OE mice exhibited a prolongation of the PR interval as early as 3-week-old (46 ± 4 vs 35 ± 2 ms, p < 0.01) (Suppl Fig. S1), and this first-degree heart block remained constant until death. Approximately 20% of the mice displayed auriculoventricular blocks (Mobitz I and II) but no third-degree AV block. No ventricular arrhythmia was recorded.

**Table 1:** Echocardiography reports:

	N	Ejection fraction (%)	Corrected LV mass (mg)	IVSd (mm)	IVSs (mm)	LVIDd (mm)	LVIDs (mm)	LVPWd (mm)	LVPWs (mm)	LV vols d (µL)	LV vols s (µL)
**Control**											
3 w	8	65.1 ± 3	44.7 ± 7	0.64 ± 0.1	0.87 ± 0.1	3.00 ± 0.4	1.96 ± 0.3	0.66 ± 0.0	0.95 ± 0.1	36.0 ± 13	12.5 ± 4
4 w	8	64.7 ± 8	44.3 ± 5	0.6 ± 0.1	0.94 ± 0.1	3.11 ± 0.2	2.04 ± 0.3	0.62 ± 0.0	0.93 ± 0.1	38.7 ± 8	13.9 ± 5
5 w	8	66.5 ± 9	50.8 ± 8	0.67 ± 0.1	0.96 ± 0.1	3.13 ± 0.2	2.00 ± 0.2	0.68 ± 0.0	1.00 ± 0.1	39.2 ± 7	13.1 ± 4
6 w	8	64.8 ± 7	65.6 ± 9	0.71 ± 0.1	1.03 ± 0.1	3.42 ± 0.1	2.23 ± 0.2	0.76 ± 0.1	1.15 ± 0.1	48.5 ± 6	17.0 ± 4
7 w	8	63.7 ± 5	67.8 ± 11	0.75 ± 0.1	1.12 ± 0.2	3.50 ± 0.1	2.32 ± 0.2	0.71 ± 0.1	1.05 ± 0.0	51.2 ± 6	18.8 ± 4
8 w	8	61.5 ± 5	81.1 ± 7	0.77 ± 0.1	1.11 ± 0.1	3.61 ± 0.2	2.43 ± 0.1	0.85 ± 0.1	1.16 ± 0.0	55.1 ± 9	20.9 ± 1
**PDZRN3 OE**											
3 w	10	67.1 ± 1	46.1 ± 7	0.64 ± 0.1	0.84 ± 0.1	2.75 ± 0.2	1.76 ± 0.1	0.72 ± 0.0	1.00 ± 0.1	28.6 ± 7	9.4 ± 2
4 w	10	59.9 ± 8	47.4 ± 6	0.64 ± 0.1	0.90 ± 0.1	3.00 ± 0.2	2.07 ± 0.1	0.71 ± 0.0	0.96 ± 0.1	34.4 ± 6	14.1 ± 2
5 w	10	51.5 ± 14	55.2 ± 9	0.67 ± 0.1	0.88 ± 0.1	3.24 ± 0.3	2.41 ± 0.4	0.71 ± 0.0	0.88 ± 0.0	43.0 ± 10	21.4 ± 9
6 w	10	44.4 ± 7*	63.3 ± 12	0.71 ± 0.1	0.93 ± 0.1	3.50 ± 0.3	2.75 ± 0.3	0.68 ± 0.1	0.78 ± 0.1**	51.6 ± 10	29.1 ± 7*
7 w	10	40.9 ± 10**	73.5 ± 11	0.73 ± 0.1	0.85 ± 0.2*	3.69 ± 0.1	2.96 ± 0.2*	0.73 ± 0.0	0.87 ± 0.1*	58.2 ± 7	34.2 ± 6**
8 w	10	39.0 ± 10**	79.9 ± 22	0.70 ± 0.1	0.84 ± 0.1*	3.72 ± 0.3	3.03 ± 0.4*	0.81 ± 0.1	0.85 ± 0.1**	60.0 ± 15	37.0 ± 12**
***P***		< 0.0001	NS	NS	< 0.0003	NS	< 0.0001	NS	< 0.0001	NS	< 0.0001

P: results of two-way ANOVA with repeated measures comparing PDZRN3 to Littermate mice group.

*< 0.05 **< 0.01; comparing at each time point PDZRN3 to Littermate mice.

IVSd and IVSs—intraventricular septum thickness in diastole and systole.

LVIDd and LVIDs—Left ventricular internal diameter and diastole and end systole.

LVPWd and LVPWs—Left ventricular posterior wall thickness in diastole and systole.

LV Vols d and LV Vols s—Left ventricular volume and diastole and end systole.

Cardiac overexpression of *Pdzrn3* led to an eccentric hypertrophic phenotype (Fig. 2c and Table 1). At 6 weeks, *Pdzrn3* OE mice exhibited an increase in the internal cross-sectional area of the left ventricle compared to their control littermates (Fig. 2d), and increased in the heart-to-body-weight ratio (Fig. 2e), and an expected increase in mRNA expression of molecular markers for cardiac remodeling, including atrial natriuretic factor (*Nppa*) and brain natriuretic peptide (*Nppb*) (Fig. 2f). As early as 6 weeks, histological analysis revealed an increase in fibrosis and inflammation in the hearts of *Pdzrn3* OE mice (Fig. 2g). Furthermore, glycogen accumulation was confirmed by the presence of periodic schiff positive materials in 4-week-old mice. We then monitored and quantified an increase in glycogen storage in situ by the Fourier Transform Infrared Spectroscopy (FTIR) method in heart tissue sections of mutant mice (Fig. 2h) at two and four weeks of age, suggesting that precocious overexpression of *Pdzrn3*, during the first weeks of life, can affect CM metabolism which may cause or precede the development of heart failure.

### Pdzrn3 signaling is required for cardiomyocyte polarized elongation along the development of heart disease in adult and postnatally

**Figure 3:**
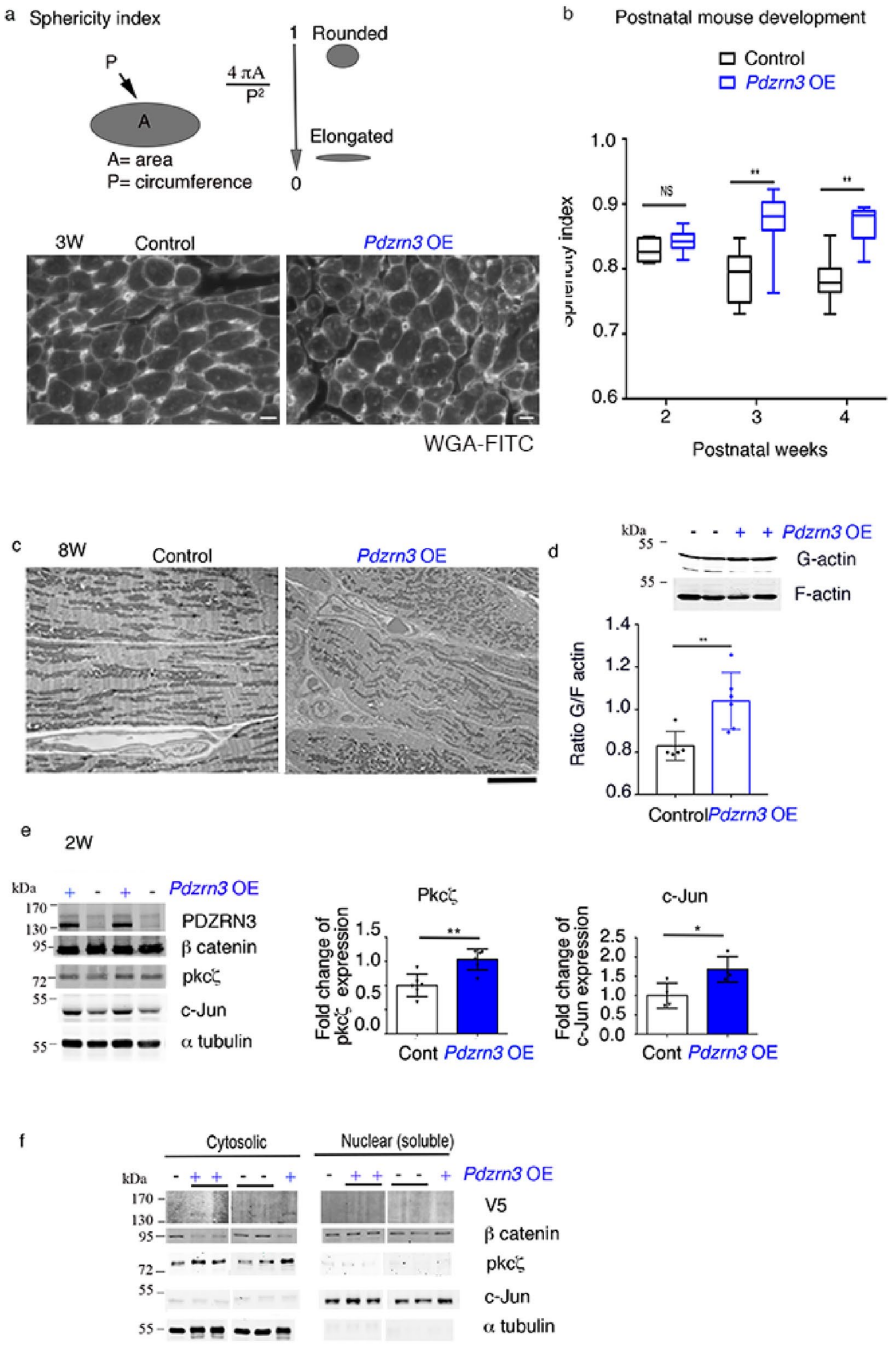
Cardiac specific overexpression of *Pdzrn3* impairs cardiomyocyte elongation. (**a**) The sphericity index was determined by measuring the area (A) and the circumference (P) of myocytes. The ratio of an “ideal” round cell is close to 1, whereas that of an elongated cell is closer to 0. Cardiomyocytes that were in the longitudinal plane were selected for quantification of the sphericity index. (**b**) Representative images of control and *Pdzrn3* OE heart sections stained with wheat germ agglutinin (WGA)-FITC (scale bars represent 20 μm) at 3 W. Quantification of myocyte cross-sectional sphericity of control and *Pdzrn3* OE heart sections. Data are represented as mean ± sem. Significance vs control **P < 0.01 by one way ANOVA plus bonferroni test (control vs. *Pdzrn3* OE group, n = 4 at 2 weeks, n = 3 at 3 weeks and n = 5 at 4 weeks). (**c**) Representative electron micrographs showing morphological disorganization of cardiomyocytes of control and *Pdzrn3* OE heart at 8 weeks of age (scale bars represent 1 μm). (**d**) Quantitative F-actin/G-actin ratios in heart lysates from 2-week-old mice were measured with actin polymerization in vivo assay kit. Western blot analysis of G-actin (G) and F-actin (F) fraction from WT and Pdzrn3 OE heart extracts were probed with anti-actin antibody. Ratios of F-actin/G actin was determined from the blots by optical density measurements. **P < 0.001, by unpaired *t*-test. (**e**) Western blot analysis of indicated proteins in heart tissues retrieved from control and *Pdzrn3* OE mice at 2 weeks (W) of age. Relative expression of Pkc ζand c-Jun from Western blots. Significance vs. control **P < 0.01; ***P < 0.001 by unpaired *t*-test (at 1 week, n = 4 mice per group; at 2 weeks, n = 6 mice per group). (**f**) Western blot analysis of indicated proteins in heart tissue retrieved from control and *Pdzrn3* OE mice after tissue fractionation to isolate cytosolic, membrane and nuclear (soluble) fractions. (n = 3 mice per group).

Cross-sectional area of CM was measured using FITC–wheat germ agglutinin–stained sections and the circularity index (*c* = 4*πSurface*/*perimeter*^2^) was followed as an index of changes in myocyte shape (Fig. 3a).

As cardiomyocyte elongation and alignment starting from the perinatal stage is a critical step in cardiomyocyte maturation, we test the hypothesis that maintaining PDZRN3 signaling during this postnatal period in mice may perturb cardiomyocyte maturation. We report that ventricular CM underwent a specific lengthening 2 to 4 weeks after birth, consistent with cardiomyocyte maturation and that forced ectopic *Pdzrn3* expression impaired shape changes in CM which remained in a round shape (Fig. 3a,b). Ultra-structure analysis (electronic microscopy) of hearts from *Pdzrn3* OE mice confirmed this analysis and revealed that cardiomyocyte loss their planar polarized organization and acquired a round phenotype at 8 weeks of age (Fig. 3c).

Pdzrn3 was known for its involvement in cytoskeletal rearrangement^9^. Because actin cytoskeleton is involved in morphogenetic process as elongation^11^, we analyzed the relationship between actin and Pdzrn3. The G/F-actin ratio significantly increased in the Pdzrn3 OE condition, suggesting an impairment or reduction in actin polymerization state in cardiomyocyte where Pdzrn3 levels were increased (Fig. 3d).

To further investigate the molecular signature correlated with impair cardiomyocyte postnatal maturation and planar polarity, we examined previously shown mediators of the PCP pathway as PKCζ and c-Jun. We found that PKCζ and c-Jun protein levels were significantly increased at 14 d in heart lysates from *Pdzrn3* OE mice (Fig. 3e). A protein fractionation strategy was then employed to separate and enrich the various cellular structures from *Pdzrn3* OE mice and control littermate tissue samples at 14 d (Fig. 3f). The amount of PKCζ protein was increased in the cytosol fraction while c-Jun was translocated in the nucleus. Total β catenin protein level was decreased in the cytosol fraction but not at the membrane. Altogether, these data suggest that PDZRN3 signaling in CM, favors c-Jun and PKCζ activation, molecular actors of the Wnt non-canonical pathway, while it impairs β catenin canonical Wnt signaling.

### Myocardial *Pdzrn3* reactivation alters postnatal cardiomyocyte maturation

**Figure 4:**
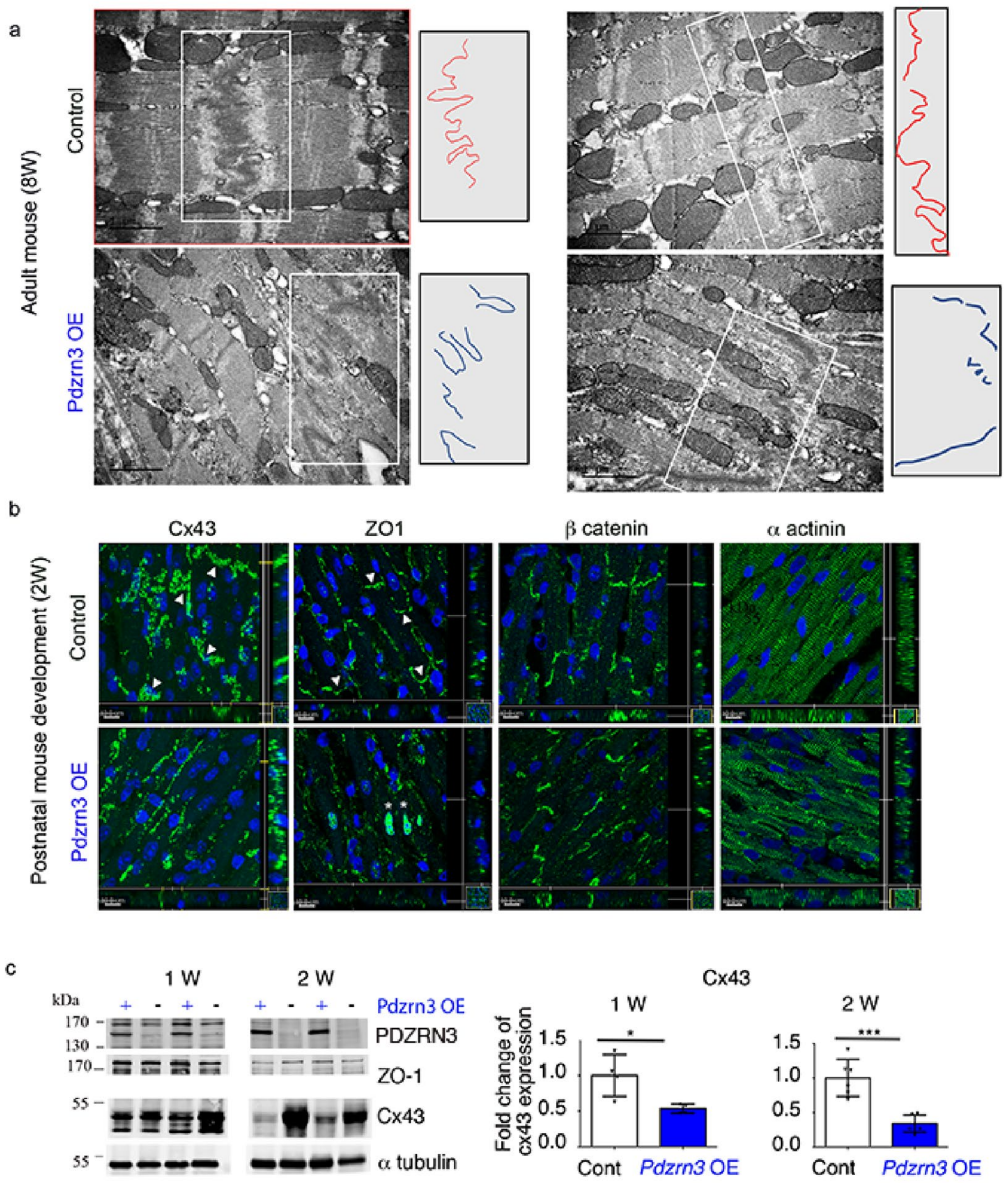
Myocardial reactivation of Pdzrn3 alters postnatal cardiomyocyte maturation. (**a**) Electron micrographs of part of an intercalated disk between two cardiac of control and *Pdzrn3* OE heart at 8 weeks of age (scale bars represent 2 μm) and in right part, schema of ID distribution. (**b**) Immunolabeling of Cx43, ZO1, β catenin and α actinin in hearts from control and *Pdzrn3* OE mice at 2 weeks. Scale bars represent 10 μm. (**c**) Western blot analysis of indicated proteins in heart tissues retrieved from control and *Pdzrn3* OE mice at 1 and 2 weeks (W) of age. Relative expression of Cx43, from Western blots. Significance vs. control *P < 0.05; ***P < 0.001 by unpaired *t*-test (at 1 week, n = 4 mice per group; at 2 weeks, n = 6 mice per group).

To identify protein biomarkers specific for impaired postnatal cardiomyocyte polarization under *Pdzrn3* ectopic expression, a mass spectrometry (MS) quantitative analysis was performed on heart lysates from *Pdzrn3* OE mice and control littermate tissue samples at 14 day. 4363 proteins were identified and among these, 39 proteins were differentially regulated, which includes 23 up-regulated and 16 down-regulated proteins (with p value < 0.05 set as significant) (Suppl Table 1). Gene ontology (GO) analyses were then carried out and we found that several proteins are functionally associated with the maintain of cell junction and cell leading edge (Suppl Table 2). Consistent with this, by electron microscopy we show a massive regression of junctions at the longitudinal cell edges between CM at 2-month-old *Pdzrn3* OE mice (Fig. 4a).

These results prompted us to hypothesize that forced *Pdzrn3* expression may impair localization of cell–cell junctional components during the initial myocyte maturation steps in mice. Henceforth, analysis of proteins that localize to intercalated disk (ID) were prioritized. First, we focused on Cx43 expression as Cx43 (*Gja1* gene) sorted out as a specific downregulated biomarker at 2 weeks in *Pdzrn3* OE mouse mutant hearts (Suppl Table 1). Immunohistological staining confirms MS analysis; the expression of Cx43 decreased as early as 1 week and was dramatically reduced at 2 weeks in heart sections of *Pdzrn3* OE mice. At 2 weeks, Cx43 expression is still detected in lateral membrane but not in ID as compared to control littermates (Fig. 4b). Western blot semiquantitative analysis confirmed the significant decrease of Cx43 expression at 7 day and 14 day (Fig. 4c).

The physiologic ZO-1 re-localization from the lateral cell membrane toward the IDs at 14 d was impaired in *Pdzrn3* OE mice compared to control littermates (Fig. 4b,c). We also found an unexpected translocation of ZO1 in the nucleus which may be correlated with a decrease of maturity of cell/cell contact^12^. At that time, we did not observe modification in expression and localization of other junctional proteins, including β catenin and N cadherin in mutant hearts (Fig. 4b and Suppl Fig. 2).

### Loss of *Pdzrn3* increases Desmoglein accumulation at the cell–cell membrane

**Figure 5:**
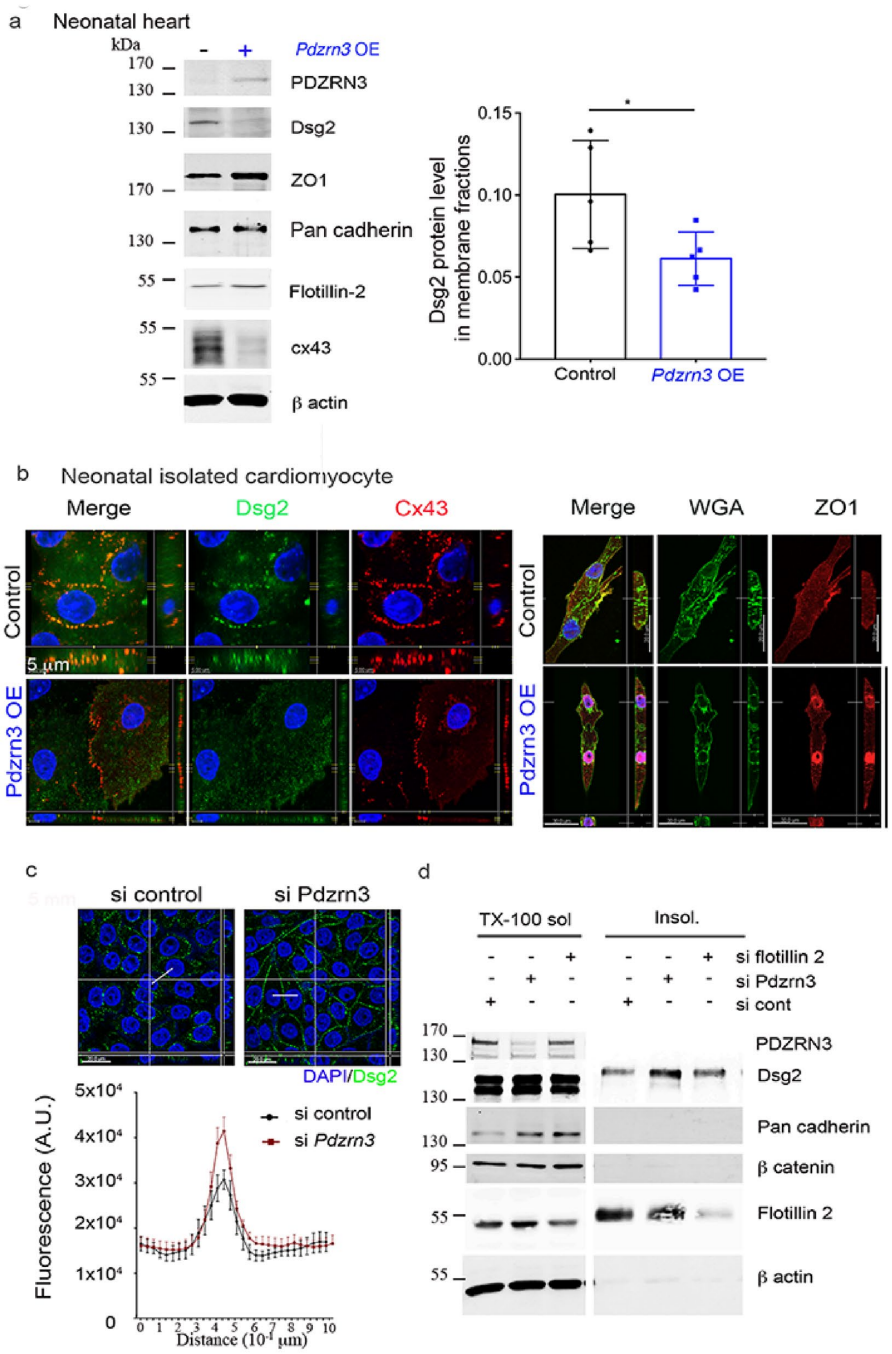
Altered subcellular distribution of junction proteins in cell–cell membrane under *Pdzrn3* induced signaling. (**a**) Western blot analysis of indicated proteins in plasma membrane fraction only of heart tissues retrieved from control and *Pdzrn3* OE mice at 2 Weeks of age. Relative expression of Dsg2, from Western blots. Significance vs. control *P < 0.05; 1 by unpaired *t*-test (n = 5 mice per group). (**b**) Representative confocal microscopy images of primary cardiomyocytes from control and *Pdzrn3* OE neonates stained with antibodies against Dsg2, Cx43, WGA-Fitc and ZO1. (**c**) After immunostaining of HeLa cells depleted of *Pdzrn3* (si Pdzrn3) or not (si control) for Dsg2 (green), fluorescence intensity profile curve were plotted. Data are represented as mean ± sem from three independent experiments. (**d**) Western blot analysis of indicated proteins in triton-X-100 soluble and insoluble fractions from HeLa depleted either of *Pdzrn3* (si Pdzrn3) or *flotillin-2* (si flotillin 2).

Alteration in adherent junction is reported to precede Cx43 gap junction changes^1,13^. We then hypothesized that *Pdzrn3* may impact on the maintain of junctional complex of cardiac muscle. The cadherin-like adhesion molecule Desmoglein (Dsg) is crucial for cardiomyocyte cohesion and is dynamically localized in ID during the first 2 weeks after birth and functions to maintain tissue integrity^14,15^. MS quantitative analysis did not show significant decrease of overall Dsg2 levels at 2 weeks in the heart lysate from mutant compared to control hearts. We then performed subcellular fractionation from the heart and revealed that Dsg2 protein levels were decreased in the plasma membrane fraction of heart lysates from *Pdzrn3* OE mice compared to control mice at 2 weeks by Western blot analysis (Fig. 5a). Using immunofluorescence analysis, we confirmed this morphological phenotype on neonatal CM isolated from *Pdzrn3* OE mice vs. control littermate mice with a decrease expression of Dsg2 and Cx43 at cell/cell junctions and a translocation of ZO1 in the nucleus (Fig. 5b). In vitro studies were then conducted to analyze whether Dsg2 accumulation at the cell membrane is regulated by *Pdzrn3*. We found that repressing *Pdzrn3* expression using siRNA increases accumulation of Dsg2 at cell/cell contact by immunofluorescent assays (Fig. 5c). Many components of cell junctions are insoluble in detergents as triton X-100 (Tx-100) due to their association with the cortical actin at the cell periphery. Dsg2 expression level increases in Triton X100-insoluble flotillin enriched membrane fraction of *Pdzrn3* depleted cells suggesting that PDZRN3 is a key determinant of Dsg2’s localization at cell–cell junctions (Fig. 5d). Overall, these findings confirm that PDZRN3-induced signaling controls the dynamic junction complex assembly at cardiomyocyte ID.

### Narrow postnatal time window is required for cardiac cell differentiation

**Figure 6:**
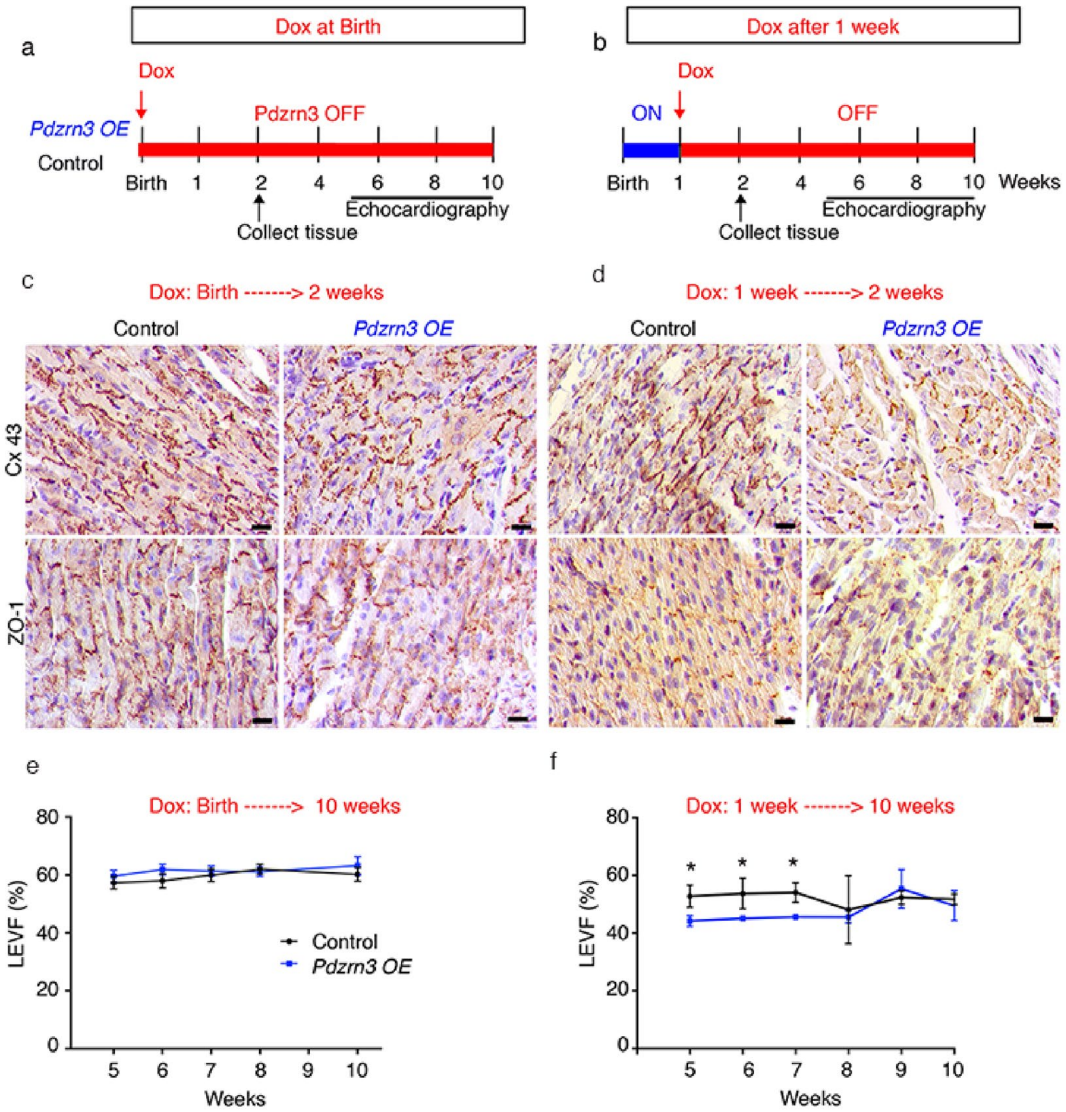
Postnatal time window for cardiac cell differentiation. (**a**, **b**) Schematic study timeline of doxycline treatment administration (Dox), tissue collection and echocardiography follow up. (**c**, **e**) Immunolabeling of Cx43 and ZO1 in hearts from control and *Pdzrn3* OE mice following doxycycline treatment. Scale bars represent 50 μm. (**d**, **f**) LVEF quantification under doxycycline treatment in control and Pdzrn3 OE mice in d protocol, respectively n = 6 vs. 8; in h protocol, respectively n = 6 vs. 4 mice.

Having shown that PDZRN3 is a critical trigger in controlling postnatal cardiomyocyte maturation, and in light of previous reports that cardiomyocyte maturation must be completed within a short time window^16^, we conducted experiments to assess whether, transgenic *Pdzrn3* overexpression within a restricted postnatal time period, is sufficient to alter cardiomyocyte maturation and to induce an eccentric hypertrophic response in adult mice. Doxycycline, a stable analog of tetracycline, was delivered to mice in the drinking water after weaning, to block *Pdzrn3* expression. Mice were administrated with doxycycline either from 0 day (birth) (Fig. 6a) or from 7 days (Fig. 6b) and sacrificed at 14 days for histological analysis. In both protocols, Western blot analysis confirmed that doxycycline treatment impaired ectopic PDZRN3 expression (Suppl Fig. S3). As expected, repression of ectopic *Pdzrn3* expression from birth abrogated the decrease of Cx43 expression and the mis-localization of ZO-1 (Fig. 6c and Suppl Fig. S3) while overexpression of *Pdzrn3* only during the first week (7–14 days doxycycline treatment) was sufficient to significantly decrease Cx43 expression and maintain ZO-1 expression in lateral cardiomyocyte membranes (Fig. 6d and Suppl Fig. S3). This data suggests that ectopic PDZRN3 signaling in the first week of life represses Cx43 expression and ZO1 re-localization.

To examine long-term effects of restricted ectopic *Pdzrn3* expression during the first week of life, cardiac function was analyzed for up to 4 months under doxycycline treatment. When *Pdzrn3* postnatal expression was repressed at birth, no signs of cardiomyopathy were detected; cardiac structure, dimension and LVEF were all normal (Fig. 6e). *Pdzrn3* OE mutant mice treated with doxycycline beginning at 7 days exhibited impaired LVEF at 8 weeks; interestingly, this alteration did not persist after 10 weeks (Fig. 6f).

Altogether, these data suggest that PDZRN3 is a critical master regulator of cardiomyocyte postnatal maturation and cardiac morphogenesis.

### Loss of *Pdzrn3* protects the transitions of the heart from compensated to decompensated cardiac hypertrophy

Reactivation of fetal gene programs have been observed in pathological processes underlying maladaptive changes in cardiac hypertrophy^17,18^, and cardiac hypertrophy to heart failure transition was reported to activate cardiomyocyte gene programs that orchestrate morphological phenotype^19^. therefore we asked whether *Pdzrn3* gene expression was related to myocardial hypertrophic adaptation.

**Figure 7:**
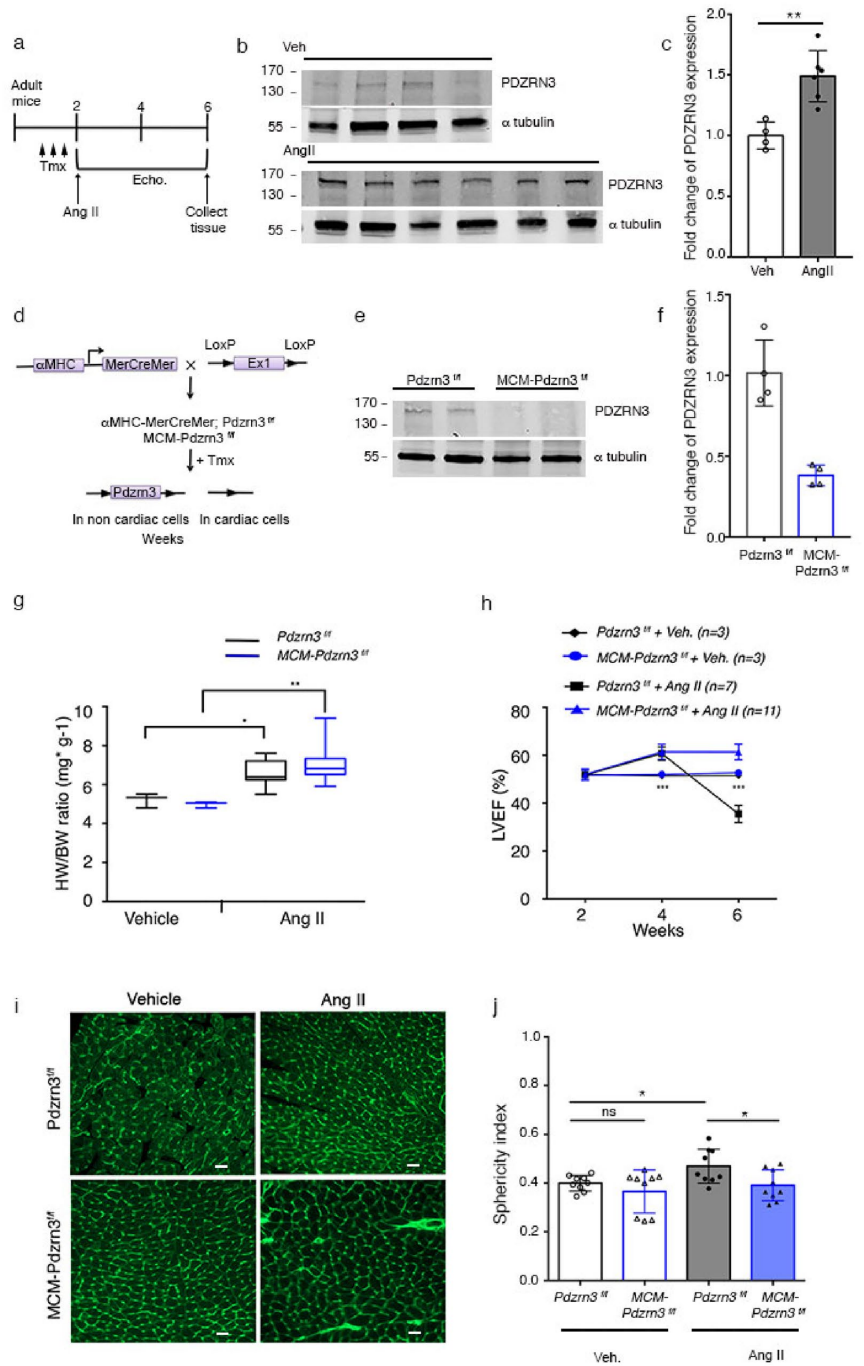
Depletion of *Pdzrn3* protects against decompensated cardiac hypertrophy. (**a**) Schematic study plan showing timeline of tamoxifen administration (Tmx), Ang II pump implantation, echocardiography (Echo.) follow up and tissue collection. (**b**) Western blot reporting levels of PDZRN3 in the adult mice under sham condition (Veh) and after AngII treatment. αtubulin was used as the loading control. (**c**) PDZRN3 ratio is quantified in veh and AngII groups and calibrated to the average of veh mice. Data are expressed as mean ± SD. Significance vs. control **P < 0.001 by unpaired *t*-test (n = 4 in veh- and n = 6 in AngII treated-groups). (**d**) Schematic of *Pdzrn3* deletion in cardiac cells. Control mice were *Pdzrn3*^*f/f*^. MCM-*Pdzrn3* KO mice were MHC-MerCreMer; *Pdzrn3*^*f/f*^. (**e**) Representative Western blot demonstrates conditional depletion of *Pdzrn3* in MCM-*Pdzrn3*^*f/f*^ mice compared to *Pdzrn3*^*f/f*^ control mice. αtubulin was used as the loading control. (**f**) The ratio is quantified and calibrated to the average of *Pdzrn3*^*f/f*^ control mice. Data are expressed as mean ± s.d. Significance vs. control *P < 0.05 by unpaired *t*-test (n = 3 per group). (**g**) Ratio of heart weight (HW) to body weight (BW). (**h**) Quantification of left ventricular ejection fraction (LVEF) in tamoxifen treated *Pdzrn3*^*f/f*^ and MCM-Pdzrn3 mice following Ang II treatment (vehicle treated groups, n = 3; Ang II-treated groups, *Pdzrn3*^*f/f*^ n = 7, MCM-Pdzrn3, n = 11). (**i**) Representative images of heart sections from f *Pdzrn3*^*f/f*^ and MCM-Pdzrn3^*f/f*^ mice following either Ang II or Veh treatment, stained with wheat germ agglutinin (WGA)-FITC (scale bars represent 20 μm). (**j**) Quantification of the cardiomyocyte sphericity index in tamoxifen treated *Pdzrn3*^*f/f*^ and MCM-Pdzrn3^*f/f*^ mice following either Ang II or Veh treatment. (n = 9 per group). *P < 0.05, **P < 0.01. One-way ANOVA with post-hoc tukey’s test.

We examined *Pdzrn3* expression a model of pressure-overload stress of the heart by Angiotensin-II (Ang II) infusion that caused cardiac hypertrophy and lead to heart failure (decompensation phase) (Fig. 7a). We found an increased expression at the transcript and protein levels for *Pdzrn3* associated with heart failure, 4 weeks post Ang II treatment (Suppl Fig. S4 and Fig. 7b,c). Here we hypothesized that Pdzrn3 re-induction was involved in CM polarization changes during hypertrophy. The loss of *Pdzrn3* may contribute to the mechanism of protecting the heart from transitions from a compensated to a decompensated cardiac hypertrophy in promoting cardiomyocyte elongation. We crossed mice bearing a *Pdzrn3* flox allele (*Pdzrn3*^*f/f*^*)* with transgenic mice (MerCreMer) that express Cre recombinase in a tamoxifen-inducible and cardiomyocyte-specific manner (Fig. 7d). The resulting MCM-*Pdzrn3*^*f/f*^ mice were indistinguishable in appearance from age-matched control *Pdzrn3*^*f/f*^ littermates. In 8 week-old MCM-*Pdzrn3*^*f/f*^ mice, treated with tamoxifen for 3 consecutive days, efficient loss of *Pdzrn3* expression at the transcript and protein levels were observed (Suppl Fig. S4 and Fig. 7e,f). There was no alteration in left ventricular ejection fraction (LVEF) and other cardiac parameters associated with *Pdzrn3* deletion as analyzed by echocardiography over 6 months (data non shown).

Interestingly, we found that tamoxifen-treated MCM-*Pdzrn3*^*f/f*^ mice are resistant to Ang II-induced maladaptive cardiac hypertrophy. Ang II infusion induced in both *Pdzrn3*^*f/f*^ and MCM-*Pdzrn3*^*f/f*^ mice development of a left ventricular hypertrophy as shown by an increase in heart-to-body weight ratios compared to both vehicle treated *Pdzrn3*^*f/f*^ or MCM-*Pdzrn3*^*f/f*^ mice (Fig. 7g). Two weeks after Ang II infusion, both *Pdzrn3*^*f/f*^ and MCM-*Pdzrn3*^*f/f*^ mice showed a small increase of LVEF. One month after Ang II treatment, *Pdzrn3*^*f/f*^ mice showed an eccentric heart hypertrophy and severe contractile dysfunction, whereas MCM-*Pdzrn3*^*f/f*^ mice maintained their LVEF at the same level as seen at earlier time points (Fig. 7h).

In adult mice treated with AngII for 4 weeks, the circularity index was significantly increased in *Pdzrn3*^*f/f*^ compared to MCM-*Pdzrn3*^*f/f*^ mice while no significant change in the sphericity index was observed in vehicle-treated *Pdzrn3*^*f/f*^ and MCM-*Pdzrn3*^*f/f*^ mice (Fig. 7i,j).

Altogether these data indicate that in CM, *Pdzrn3* is essential in the transition from concentric to eccentric hypertrophy and subsequent heart failure in models of induced cardiac hypertrophy.

## Discussion

Both canonical and non-canonical Wnt signaling have been implicated in the multistep process of cardiogenesis^20–22^, however, understanding of the outcome of activating and inhibiting the Wnt pathways at specific times during the development of the heart is still not fully understood. In normal adult CM, Wnt/Fzd is quiescent and becomes reactivated in the development of pathology such as hypertrophy^23^. The stabilization of β catenin induces hypertrophic growth^24^ while β catenin cardiac deletion attenuates TAC-induced hypertrophy^25,26^. It was reported that JNK overexpression, in vivo*,* led to a lethal cardiomyopathy^27^.

### PDZRN3 and non-canonical signalling

Our data seems to favor the hypothesis that in the heart, PDZRN3 is acting in the PCP pathway. There is a lack of consensus regarding the biochemical nature of Wnt non-canonical pathway regulation, and variability in the methods used to measure signaling and effects. Previously, we have shown that PDZRN3 drive non-canonical Wnt signaling and interacted with the polarity proteins PAR3, PKC, and MUPP1 at endothelial cell junctions to regulate tight junction stabilization, polarized migration and vascular morphogenesis^9^. A recent work reported that activation of Wnt5a-Ror signaling results in the regulation of Pdzrn3 abundance in a Wnt/β-catenin independent manner, mediated by a signaling cascade involving Fzd receptors, Dvl scaffolding proteins, GSK3, and CK1 that culminates in UPS-dependent degradation of Pdzrn3^10^. Here, in CM, we report that PDZRN3 overexpression, in the first 2 weeks after birth, leads to an increase of PKCζlevels with translocation of c-Jun in the nucleus, which correlated with a decrease of cytosolic ß catenin without modification of the membrane ß-catenin pool. This is consistent with reports showing a role of the non-canonical Wnt pathway during cardiogenesis which destabilizes ß catenin dependent canonical Wnt signals^28^. We also demonstrated that Pdzrn3 induces cortical actin rearrangement by regulating actin-associated proteins, myosin light chain kinase, in cultured ECs^9^. Because actin cytoskeleton is involved in morphogenetic process as elongation^11^, we analyzed the relationship between actin and Pdzrn3. The G/F-actin ratio significantly increased in the Pdzrn3 OE condition, suggesting an impairment or reduction in actin polymerization state in cardiomyocyte where Pdzrn3 levels were increased.

Forced PDZRN3 expression impairs postnatal cardiomyocyte elongation, and final geometry, maintaining cardiomyocyte in a round shape. Conversely, PDZRN3 suppression, maintained cardiomyocyte elongation against hypertrophic stress that led to an increase in sphericity (and block heart failure evolution). Effects of PDZRN3 signaling on morphogenesis cannot be replicated in vitro on neonatal CM or immature hiPSC-derived CM because of the impaired or incomplete cell–cell contacts and 3D structure organization. However, we provide a fair correlation between PDZRN3 level and cardiomyocyte morphogenesis. In line, we showed that forced PDZRN3 expression disrupted IDs with a decrease of Dsg2 accumulation at cell borders which coincide with a low to undetectable Cx43 expression, displacing ZO-1 toward nuclei, and this was concomitant with an impaired postnatal cardiomyocyte elongation, PDZRN3 effects on cardiomyocyte junction was consistently confirmed by proteomic analysis. We show here that Pdzrn3 controls a genetic program essential for heart maturation and for the maintenance of overall cardiomyocyte geometry and contractile function. It has previously been reported that during neonatal heart maturation, and perinatally, ZO-1 becomes gradually restricted to the ID of the myocytes, where it recruits Cx43 from the initial circumferential plasma membrane to the ID to generate functional gap junction channels^29,30^. Similarly, our results in mice indicate that during the brief period after birth, neonatal CM evolve from a round, non-polarized immature state to an elongated, aligned and highly polarized state with the cell junction proteins Cx43 and ZO-1, located to the ID, joining cells end to end. Forced postnatal expression of PDZRN3 impaired Cx43 induction and dynamic localization of both Cx43 and ZO-1 at the IDs, as early as 1 week after birth, a time during which Cx43 gap junction plaques form at both cell ends. We report an almost complete loss of Cx43 staining after 15 d of *Pdzrn3* OE in transgenic hearts, which could be due to Cx43 degradation through lysosomal and proteasomal ubiquitin dependent pathways^31^. This remodeling of gap junctions may account for the reported cardiac conduction alterations in Pdzrn3 OE mice as previously reported^32^. Reduction of about 90% in Cx43 expression resulted in about 50% decrease in the conduction velocity. While 50% reduction in Cx43 may lead to some conduction slowing, high levels of electrical uncoupling are needed to increase arrhythmogeneity^33,34^.

Formation of desmosome precedes gap junction channel^35^. Dsg2 is a major and essential cadherin of the cell–cell contact in cardiac desmosomes^36^ where it helps to maintain planar coordination of cardiac myocytes. Disruption of adhesive integrity via mutations in the *Dsg2* gene is regarded to cause severe arrhythmogenic cardiomyopathy^14,37,38^. In contrast, induced deletion of Cx43 in the adult heart did not affect the spatial organization of adherens junction and desmosome in the ID^39^. In agreement, we observed a loss of cardiac tissue integrity with a rupture of cardiomyocyte junctions at the ultrastructural level in mutants overexpressing *Pdzrn3*. We showed here that forced expression of *Pdzrn3* did not modify significantly overall cellular levels of Dsg2 but diminished Dsg2 localization at the cell membrane while repressing *Pdzrn3* was sufficient to enhance Dsg2 accumulation at the cell/cell contacts. Dsg2 is dynamically localized to the plasma membrane, through a microtubule dependent transport; altering this mechanism results in weakened adhesive strength^40^. Thus, our findings suggest that PDZRN3 signaling regulates this active process of Dsg2 recruitment at the cell membrane.

In the heart, polar accumulation of junction proteins at the IDs is necessary for terminal differentiation of myocytes and is required for global adaptive architecture of the heart against hypertrophic stress. Alteration of the organization and molecular composition of contact sites between CM, the intercalated disks was related to being associated with heart failure^41–43^. We report that cardiomyocyte specific *Pdzrn3* deletion, protects the heart from the transition to heart failure, under conditions of pressure overload stress. Consistently, *Pdzrn3* sustained expression in CM during the first 2 weeks after birth, lead to abnormal heart morphogenesis, eccentric cardiac hypertrophy and early death. Previous reports have demonstrated a decrease of Cx43 with a reduction in size and abundance of gap junctions in cardiac tissues retrieved from patients with ischemic, or dilated cardiomyopathy or in end-stage heart failure^44^. Moreover, ZO-1 levels were reported to be increased at the IDs in these patients, and negatively correlated with Cx43 levels at ID. Other groups have found that ZO-1 and Cx43 levels were reduced concomitant with a reduction in co-localization between these two proteins, at IDs in humans with end-stage heart failure^45,46^*.* Additionally, the lateralization of Cx43 to adherens junction-sparse regions of the membrane has been reported in tissue from the myocardial infarct border and zones of myofiber disarray in patients with hypertrophic cardiomyopathy^47^. The lateralization of Cx43 observed in many pathological scenarios is reminiscent of arrangements observed during maturational growth of the ventricle. In this pathological recapitulation of developmental patterns in the adult, cell–cell adhesion junctions appear to remain prominently located at the ID, whereas Cx43 distributes to lateral sarcolemma^48^. During atrial dilation or permanent atrial fibrillation, there are a number of de-differentiated myocytes with lateralized connexins, altered ID and loss of polarity^49,50^. We propose that upregulation and maintenance of *Pdzrn3* and decrease of Cx43 expression may lead to a maladaptive response to hypertrophy. Although we acknowledge that mechanism and signaling pathway by which Pdzrn3 controls cardiomyocyte polarization and elongation is not fully described in this report, because of the difficulties to work on elongation and ID on an in vitro model, this study identifies PDZRN3 as a novel signaling regulator of polarized myocyte organization and as a potential therapeutic target for the prevention of human heart failure.

In conclusion, our data expands the current knowledge regarding early determination of cardiac tissue development and breaks the concept of a novel signaling pathway that controls a genetic program essential for heart maturation and maintenance of overall geometry, as well as the contractile function of CM.

## Materials and methods

### Experimental animals

Animal experiments were performed in accordance with the guidelines from Directive 2010/63/EU of the European Parliament on the protection of animals used for scientific purposes and approved by the local Animal Care and Use Committee of the Bordeaux University CEEA50 (IACUC protocol #8792). General surgical procedures were conducted in mice according to ARRIVE guidelines (https://arriveguidelines.org).

For cardiomyocyte *Pdzrn3* overexpression (Pdzrn3 OE mice), *MHC-tTA* males (Tet-off) were mated with *Pdzrn3-V5* mutant females^9^ which results in the generation of mice with genotypes of *MHC-tTA*; *Pdzrn3-V5* (Pdzrn3 OE) and Pdzrn3-V5 (control). Tails of pups were genotyped by PCR using the P1 and P2 primer set to detect the *Pdzrn3-V5* coding gene (P1, 5′-CAGCTTGAGGATAAGGCGCT-3′; P2, 5′-CTTCGAGCTGGACCGCTTC-3′) and using the P3 and P4 primer set to detect the *tTA* coding gene (P3, 5′-GCTGCTTAATGAGGTCGG-3′; P4, 5′-CTCTGCACCTTGGTGATC-3′.

For cardiomyocyte–specific deletion, αMHC-MerCreMer transgenic mice^51^ were crossbred to *Pdzrn3*^*f/f*^ mice^9^, which results in the generation of mice with genotypes of αMHC-MerCreMer; *Pdzrn3*^*f/f*^* (MCM- Pdzrn3*^*f/f*^*) and Pdzrn3*^*f/f*^*.* For *Pdzrn3* gene deletion in adults, 0.5 mg of tamoxifen was injected intraperitoneally for three successive days, 2 weeks before surgery. Doxycycline was administrated in the drinking water (0.4 mg/mL) to inhibit cardiac specific expression of transgenic *Pdzrn3*.

Isolation of neonatal CM: at P3, mice were euthanized by decapitation. Hearts were carefully removed from the thoracic cavity, immediately placed into the ice-cold cardioplegic solution. Ventricular neonatal CM were then isolated and prepared as recommended (Pierce™ primary cardiomyocyte isolation kit).

### Experimental protocols

The mouse model of cardiac hypertrophy: tamoxifen induced *Pdzrn3*^*f/f*^* and MCM- Pdzrn3*^*f/f*^ mice*,* 8 weeks old, were subjected to Angiotensin (Ang) II by the use of subcutaneously implanted miniosmotic pump (Alzet) for 4 weeks filled with either Ang II (125 ng/kg/min) or saline solutions.

Echocardiography: Mice were anesthetized using 1.5% oxygenated isoflurane by inhalation. Echocardiography was performed using a Visualsonics Series 2100 high-resolution imaging system with a 38 MHz Microscan transducer probe. Cardiac ventricular dimensions and fractional shortening were measured in 2D mode and M-mode 3 times for the number of animals indicated.

Electrocardiography: Electrocardiograms were recorded from mice sedated with low dose of isoflurane using the standard four limb leads. Waveforms were recorded with AD instruments Animal Bio Amps ECG and intervals were measured manually using Powerlab device and LabChart Pro V8 software^52^.

### RNA preparation and quantitative PCR

Mouse tissues were homogenized in TRI-REAGENT ™ (Euromedex) and RNA was extracted according to the manufacturer’s instructions. Q-PCR was performed as described previously (*19*^*9*^). The following primer sets were used: mouse cyclophyllin (NM_009505), F: 5′-AGCTAGACTTGAAGGGGAATG-3′, and R: 5′-ATTTCTTTTGACTTGCGGGC-3′; mouse *Nppa* (NM_008725), F: 5′-CGTCTTGGCCTTTTGGCTTC-3′, and R: 5′-GGTGGTCTAGCAGGTTCTTGAAA-3′; mouse *Nppb* (NM_008726), F: 5′-AAGCTGCTGGAGCTGATAAGA-3′, and R: 5′-GTTACAGCCCAAACGACTGAC-3′; mouse *Pdzrn3*, F: 5′-CTGACTCTTGTCCTGCATCGGGACTC-3′, and R: 5′-ATGGGC TCCTTGGCTGTCTTGAAAGC-3′, mouse *Gja1* (NM_010288), F: 5′-ATCAGGGAGGCAAGCCATGCTCA-3′, and R: 5′-ACGTTGGCCCACACCACAAAGA-3′. Target gene expression was normalized to that of the control gene, and relative expression was quantified by the comparative Ct method (2^−∆∆Ct^).

### Histology and immunohistochemistry

For tissue immunohistology analysis, hearts were fixed in paraformaldehyde (PFA) 4%-PBS, then paraffin embedded and sections were stained with hematoxylin/eosin for morphological evaluation, picrosirius red for fibrosis quantification and labeled with tetramethyl rhodamine isotiocyanate-labeled wheat germ agglutinin (WGA, Sigma-aldrich) for the cross-sectional area.

On each section, circularity index was calculated, *c* = 4*πSurface*/*perimeter*^2^ is the 2D equivalent of the true sphericity index. This indice ranges from 1 (perfect sphere or circle) to 0 (elongated shape). Captured images using a Zeiss (Axioimager) were analyzed with the NIH ImageJ software (Version 2.1.0) software^53^.

Immunofluorescence staining was performed as previously described^9^ with antibodies specific for CD45 (BD pharmingen), Connexin 43 (Sigma), ZO1 (Invitrogen), beta-catenin (sigma-aldrich), N Cadherin (Santa Cruz). Images were taken with a Zeiss LSM700 confocal laser-scanning microscope confocal microscope.

Transmission electron microscopy (EM) was performed on glutaraldehyde-perfused hearts as previously described^9^.

### FTIR analysis

Formalin fixed hearts were then snap frozen. A 20 µm section was then deposited onto an IR-transparent ZnS window (Crystan, UK) for FTIR analysis. Four sections from the left ventricle were analyzed. For each section, about 50 IR spectra were recorded. IR spectra were collected in transmission mode using a Spotlight 300 FTIR imaging system, coupled to a Spectrum One spectrometer (Perkin-Elmer). Data analyses were carried out with the OPUS 7.2 software subroutine (Bruker, Germany)^54^. Pre-processing of baseline correction and normalization spectra were applied. Then spectra were used for classification (hierarchical cluster analysis) using Ward’s algorithm. Classifications were performed on the second derivative of the spectra with 7-points smoothing using 1200–900 cm^−1^ spectral interval from the IR spectra. Then the average of the IR spectra from a cluster was performed and the average was compared^55^.

### F/G actin ratio measurements

The amount of F-actin and G-actin was measured with an actin polymerization assay kit (BK037, Cytoskeleton). Two-week-old mice were sacrificed by cervical dislocation. For each sample, 2 hearts were sliced into small pieces with a scalpel and quickly homogenized in 1 mL of F-actin stabilization buffer with ATP and protease inhibitors with a Dounce homogenizer and then incubated at 37 °C for 10 min. Tissue homogenates were spun at 100,000×*g* for 1 h at 37 °C to separate the globular (G)-actin (supernatant) and filamentous (F)-actin fractions. The pellets were resuspended to the same volume as the supernatant in using ice-cold Milli-Q water and 10 μM cytochalasine D (F-depolymerizing solution) and left on ice during 1 h. Actin was quantified by Western blot using an anti-actin antibody (Cytoskeleton). The F-actin/G-actin ratio was determined using the Odyssey infrared imaging system (LI-COR Biosciences).

### Cell culture and transfection

HeLa cells were cultured in RPMI supplemented with 10% fetal bovine serum and penicillin streptomycin. SiRNA were transfected using Interferin (Polyplus) at a final concentration of 30 nM. The oligonucleotides used were designed by Origene for h-pdzrn3^9^.

For subcellular triton soluble and insoluble fractions, the cells were scraped with a plastic blade in 10 mM Tris pH 7.5, 140 mM NaCl, 5 mM EDTA, 2 mM EGTA and 1% Triton X100 supplemented with 0.1 mM sodium orthovanadate, 0.1 mM PMSF, 20 μg/mL leupeptin, benzonuclease, 50 μg/mL pepstatin, aprotinin, trypsin inhibitor and 0.1 μM okadaic acid. Culture dishes then the mixture was incubated for 10 min on ice and centrifuged for 5 min at 800×*g*. The supernatant was then centrifuged for 30 min at 14,000×*g*. The supernatant was saved (Triton-X100-soluble fraction) and the pellet was dissolved in 10 mM Tris pH 7.5, 8 M urea, 140 mM NaCl, 5 mM EDTA, 2 mM EGTA and 1% SDS (triton-X100 insoluble fraction).

### Western blot and fractionation

Immunoblotting were performed as described previously^9^. For tissue fractionation, tissue lysates were processed with cell extraction kits (Thermo scientific Pierce) as recommended. Briefly, cellular compartments are sequentially extracted by incubating cells with cytoplasmic, membrane and nuclear protein buffers.

Proteins were then resolved by SDS-polyacrylamide gel electrophoresis and blotted with antibodies specific for V5 (Invitrogen), PDZRN3 (Santa Cruz), and PDZRN3 (gift from Henry Ho CA, USA). Laboratory ZO1 (Invitrogen), β-catenin (Sigma-Aldrich), PKCζ (Santa Cruz Biotechnology), c-Jun (Upstate), Cx43 (Sigma) α-tubulin (Sigma-Aldrich), Dsg2 (Acris Origene), pan cadherin (Sigma), Flotillin-2 (Santa Cruz). Binding of antibodies to the blots was detected using the Odyssey Infrared Imaging System (LI-COR Biosciences).

For mass spectrometric analysis and protein identification, proteins recovered within V5 immunoprecipitates were excised from colloidal coomassie blue-stained gels. Peptide sample preparation and LC–MS/MS analysis was performed in collaboration with the proteome platform of the Functional Genomic Center of Bordeaux (CGFB). Briefly, the excised gel pieces were destained, reduced with DTT and alkylated iodoacetamide. Excess reagents were washed out and trypsin in-gel digestion was performed. Tryptic peptides were extracted from gel plugs, dried and resuspended in 2% acetronitrile with 0.05% trifluoroacetic acid (TFA). The LC–MS/MS measurements of peptide solutions were carried out on Q-Exactive mass spectrometer (Thermo Fisher Scientific). Samples were injected on a C18 precolumn (Acclaim PepMapTM) and further separated on a 75 µm ID × 15 cm nanoviper C18 reversed phase column (Acclaim PepMap RSLC) with gradients from 4 to 40% ACN in 0.1% formic acid for 120 min min at a flow rate of 300 nl/min. Full MS scans were acquired in the Orbitrap mass analyzer over m/z 300ñ2000 range.

### Statistical analysis

The experimental results represent means ± sem. Each experiment was conducted at least three times. When multiple experiments using different numbers of animals were pooled for the statistical analysis, the range of number of animals was indicated in the figure legend. Comparison of continuous variables between two groups was performed using the unpaired two-sided Mann–Whitney *U*-test (nonparametric). Comparison of multiple groups was performed by ANOVA. Cumulative survival data was evaluated by Kaplan–Meier nonparametric regression analysis and the log-rank test. All analyses were performed with GraphPad Prism v8.0.2 (GraphPad, San Diego, California, USA) software^56^. *P* < 0.05 was considered statistically significant. The statistical test is indicated for each data analysis.

### Preprint

A previous version of this manuscript was published as a preprint 10.1101/2020.07.29.226597.

## Supplementary Information

Supplementary Information.
