# Supplementary material for: Decrease of Pdzrn3 is required for heart maturation and protects against heart failure
Source: Sci Rep. 2022 Jan 7;12:8. doi: 10.1038/s41598-021-03795-7 (PMC8742099; doi:10.1038/s41598-021-03795-7)
Supplement: Supplementary file 1 — Supplementary Information. [file 41598_2021_3795_MOESM1_ESM.pdf]

Supplementary Information file

Mathieu Pernot[1], Béatrice Jaspard-vinassa[1¶], Alice Abelanet[1¶], Sebastien Rubin[1], Isabelle Forfar[1], Sylvie Jeanningros[1], Laura Cetran[1], Murielle Han-Yee Yu[1], Elise Balse[2], Stéphane Hatem[2], Pascale Dufourcq[1,3], Thierry Couffinhal [1,4,¶¶], Cécile Duplàa[1 ¶¶] *.

Affiliations :

1. Univ. Bordeaux, Inserm, UMR1034, Biology of Cardiovascular Diseases, F-33600 Pessac, France
2. Faculté de Médecine, Université Pierre et Marie Curie, Sorbonne University, INSERM UMR_S1166, Paris, France
3. Service de Biochimie clinique, CHU de Bordeaux, Bordeaux, France.
4. Service des Maladies cardiaques et vasculaires, CHU de Bordeaux, Bordeaux, France.

¶ and ¶¶: These authors contributed equally to this work

* To whom correspondence should be addressed.

Cécile Duplàa – Inserm U1034 – 1 avenue de Magellan – 33600 PESSAC – France - Tel number: (33) 5 57 89 19 71; Fax number: (33) 5 56 36 89 79; Email: cecile.duplaa@inserm.fr

Suppl Table 1: List of biomarkers down and up regulated at 14d in heart lysate from Pdzrn3 OE mice versus littermate mice

Accession number	GENE NAME	LOG FOLD CHANGE	CORRECTED P-VALUE
Q6NVD0	Frem2	-1.50983600447357	0.0407753509995338
Q5DTX6	Jcad	-1.51708512270693	0.0405886219084547
A0A2I3BR81	Nt5dc2	-1.6022060553698	0.0239124198426907
Q0P678	Zc3h18	-1.63030820834963	0.0210791958789641
Q4VBE8	Wdr18	-1.7252593922209	0.00998035563693598
A2AQZ2	Phyhd1	-1.75156415729004	0.0085217594845209
P97414	Kcnq1	-1.76219530639184	0.00816130708455465
Q6XPS7	Tha1	-1.78718498343948	0.0072485043136236
Q8BGT5	Gpt2	-1.79793930772569	0.00712772446377335
Q8CH09	Sugp2	-1.82005004531569	0.00648706202954506
P23242	Gja1	-1.95745579903143	0.00234771751670759
A2A517	Cyth1	-1.96923657674321	0.00233273558913082
P43883	Plin2	-2.17156592723524	0.000534221640867139
O88958	Gnpda1	-2.41190592980274	6.3715662932106e-05
Q8BVZ1	Plin5	-2.91073675004248	1.66979621965998e-07
E9QLJ0	Cmya5	-3.22605931030087	3.31109041005517e-09
Q5SVT2	Trim16	1.71396242055331	0.0407753509995338
P09528	Fth1	1.72153190478464	0.0405886219084547
J3QSN2	Usp13	1.73275355351494	0.0394005005341385
O08601	Mttp	1.74326316159894	0.0374284486817251
A2ARP8	Map1a	1.79682963878194	0.0254265981829681
A0A087WRT4	Fat1	1.80980661347015	0.0239124198426907
Q8BLN5	Lss	1.81403668902523	0.0239124198426907
Q8BHT6	B3glct	1.95443844414465	0.0085217594845209
Q62009	Postn	1.97491659225251	0.00804277011869158
Q9Z1T2	Thbs4	1.99184964803742	0.0072485043136236
Q9QVP9	Ptk2b	2.01369697676567	0.00688769876934199
Q80YX1	Tnc	2.0677915841686	0.0047308563339104
O70309	Itgb5	2.0714825949603	0.0047308563339104
H7BWY6	Rbp4	2.12585277404207	0.00313336430831344
A0A140LHU0	Adam9	2.17173725207092	0.00233273558913082

Supplementary Table 1: List of identified biomarkers down and up regulated at 14d in heart lysate from Pdzrn3 OE versus littermate mice.

Supplementary Table 2

a

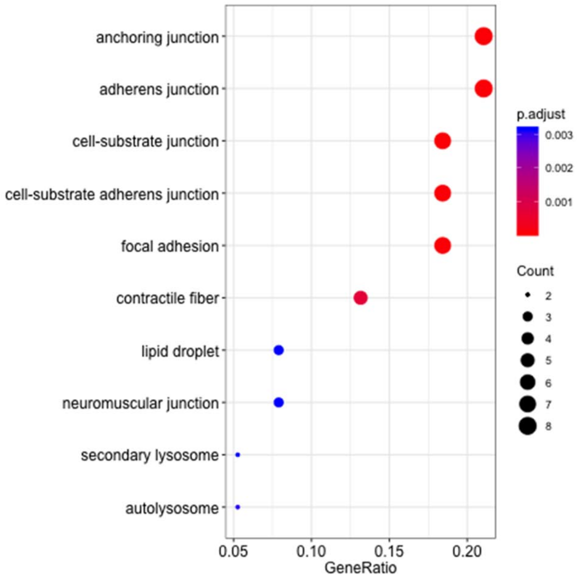

b

	Cellular component	Protein
GO:0005912	adherens junction	Gja1 Itgb5 Xirp2 Ptk2b Tnc Jcad Adam9 Fat1
GO:0031252	cell leading edge	Itgb5 App Ptk2b Jcad Fat1

Supplementary Table 2: subcellular functional classification of the identified proteins: a) hierarchical comparison of proteins grouped according to their similar subcellular locations and expressed as number of assigned proteins (P<0.05) (b) List of identified biomarkers in adherens junction and cell leading edge pathways.

Supplementary figures

Suppl Fig. S1

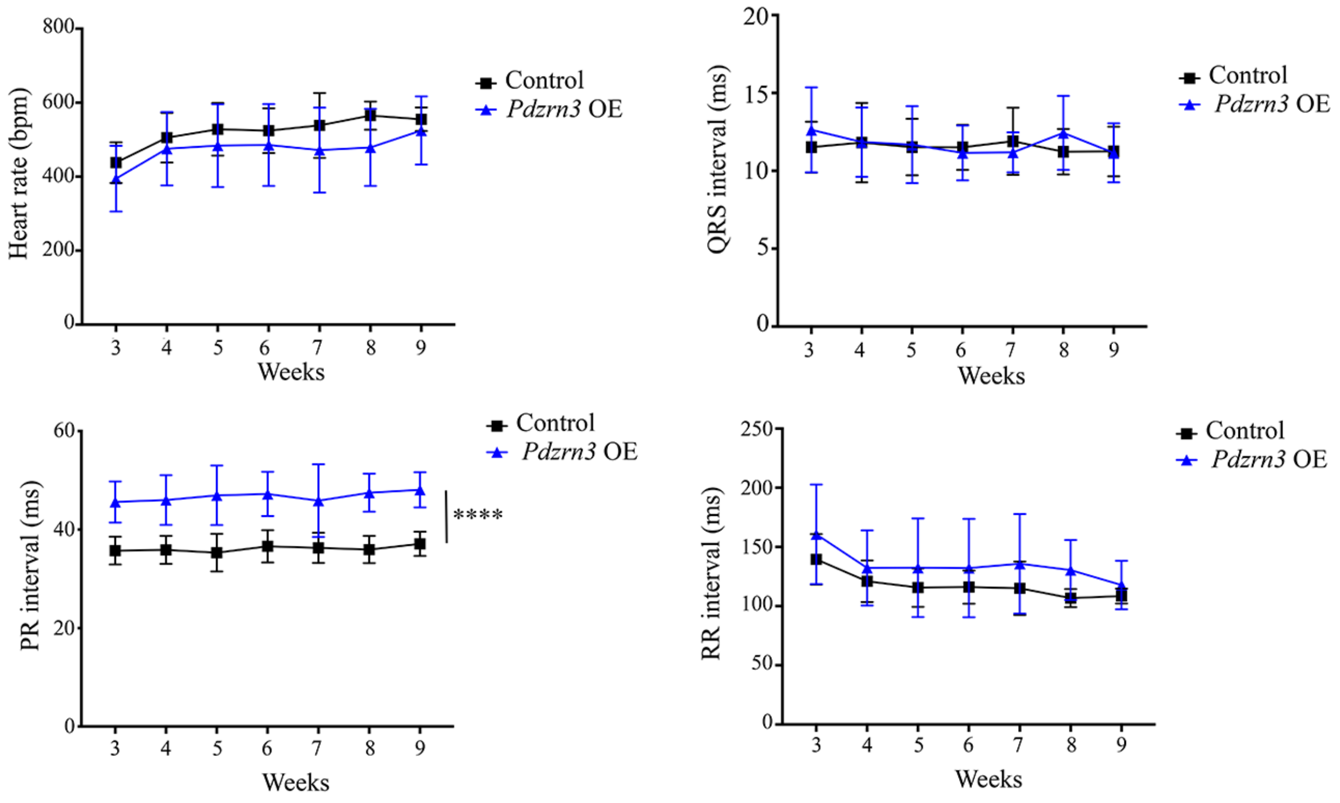

Supplementary figure S1:

Variation of heart rate (beat per min), as well as PR, QRS, and RR intervals (ms) in control and Pdzrn3 OE mice from 3 to 9 weeks after birth. (respectively n=6 mice vs. n=4 mice)

***, $P<0.001$ by repeated-measures two way ANOVA with tukey's test.

Suppl Fig S2

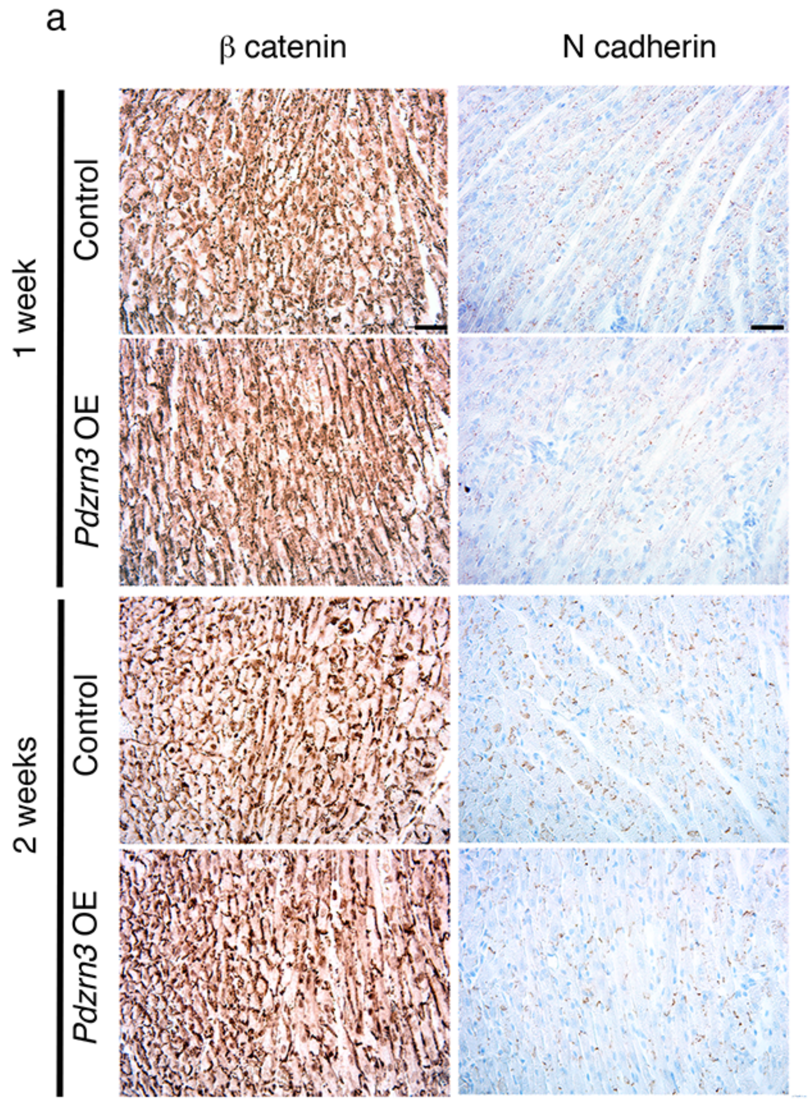

Supplementary figure S2: Immunolabeling of β catenin and N cadherin in control and *Pdzrn3* OE hearts at 1 and 2 weeks of age. Scale bars represent 50 μm.

Suppl fig S3

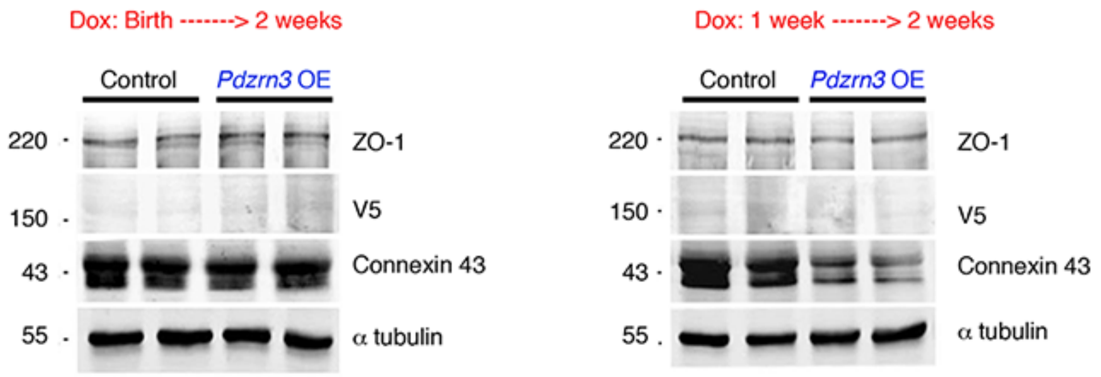

Supplementary Fig. S3: Western blot analysis of indicated proteins in heart tissues from control and *Pdzrn3* OE mice under doxycycline treatment at 2 weeks.

Suppl fig S4

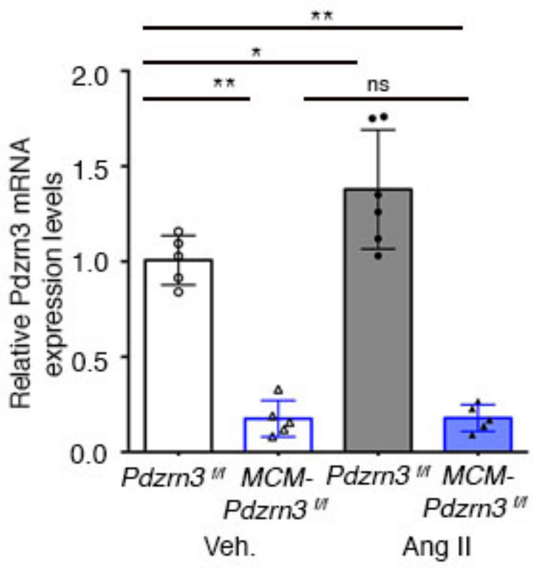

Supplementary Fig. S4: Quantitative real-time PCR analysis of Pdzrn3 transcript abundance in hearts retrieved from MCM-*Pdzrn3* KO (MCM-Pdzrn3 $^{f/f}$) and wild type mice (Pdzrn3 $^{f/f}$) after treatment with saline (Veh.) or Ang II for 4 weeks. mRNA levels were normalized to cyclophilin and are expressed as relative expression over levels in the control Veh. treated group (n=5-6 per group). *, P<0.05, ** P<0.01. Mann-Whitney test

UNCROPPED FILES

Suppl Fig. S5:

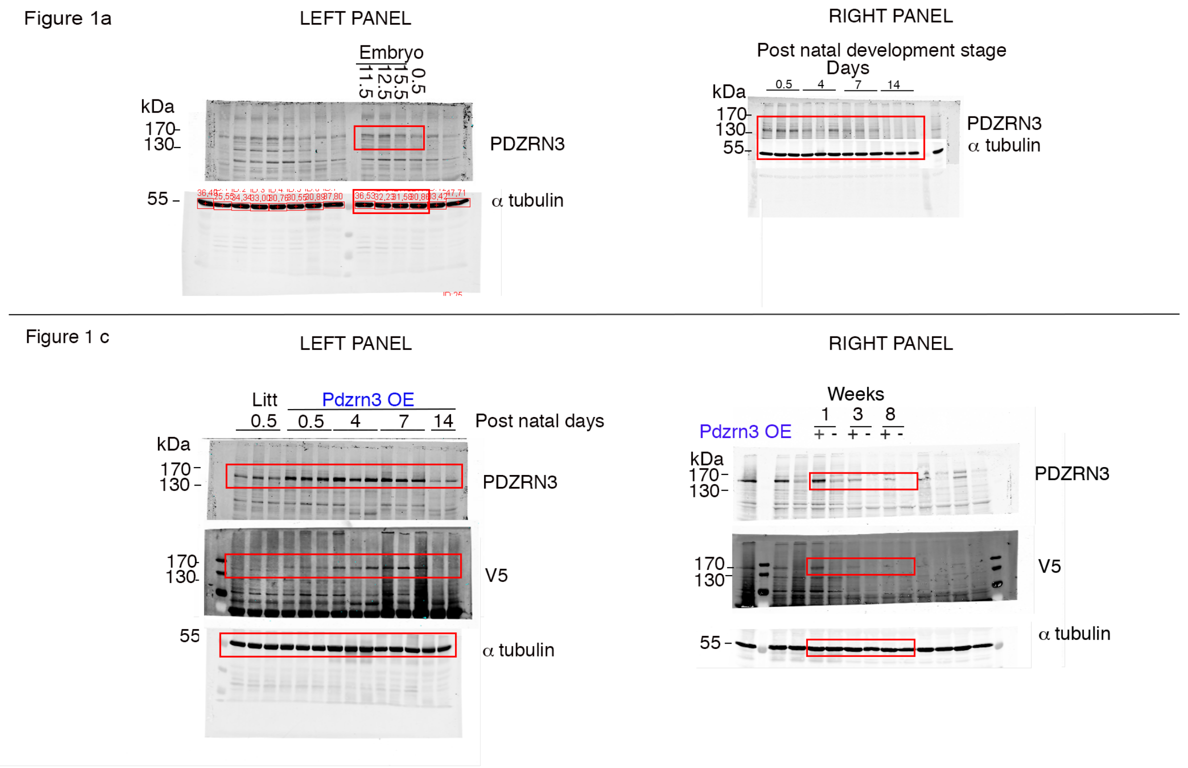

Supplementary Fig. S5:
Original images of western blotting used for figure 1a and 1c (shown as cropped images in figure 1a and 1c).

Suppl fig. S6:

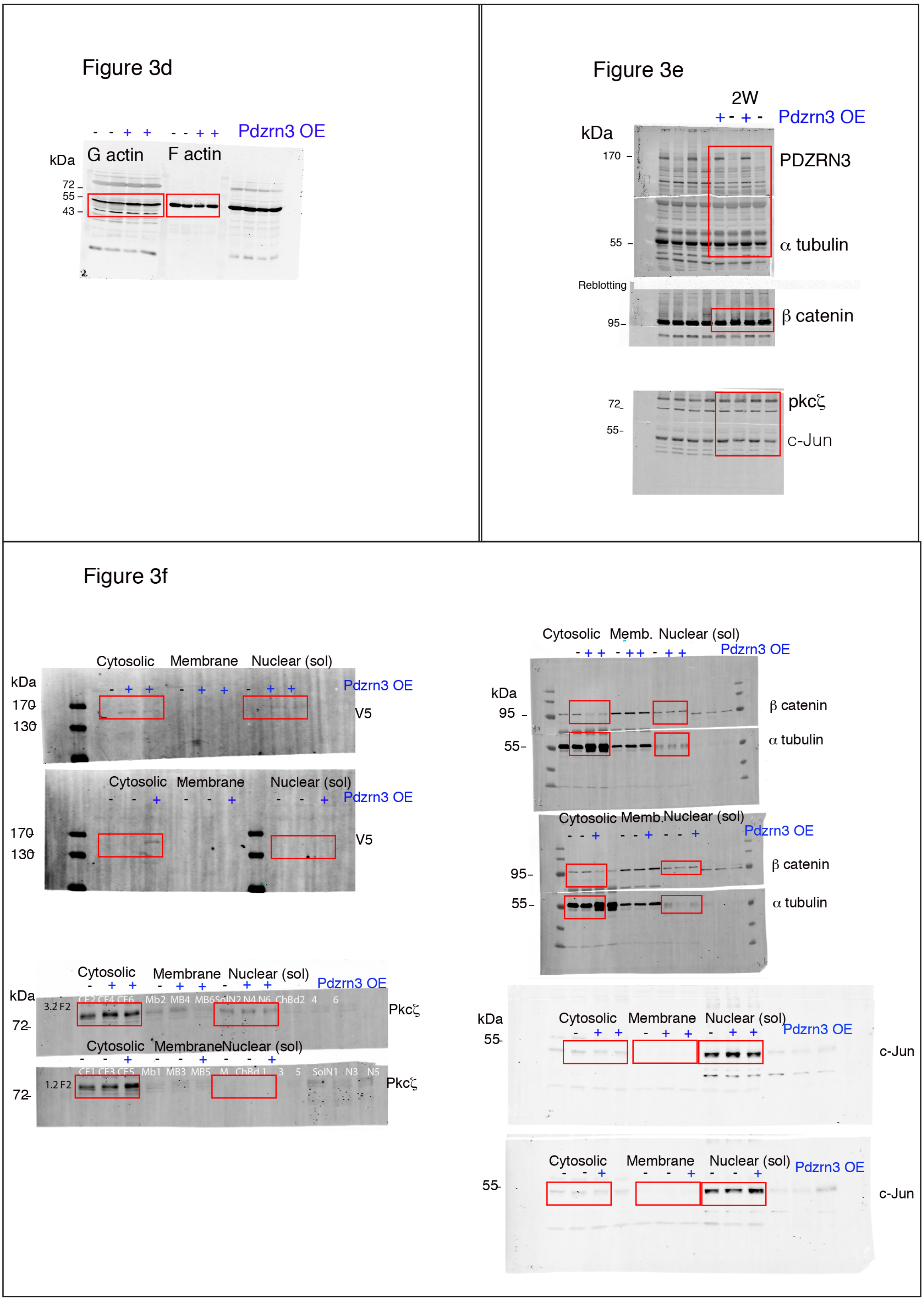

Supplementary fig. S6

Original images of western blotting used for figure 3d and 3e and 3f (shown as cropped images in figure 3).

Suppl figure S7:

Figure 4c

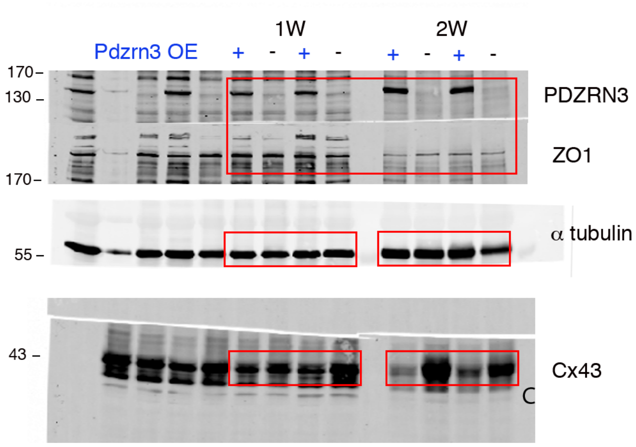

Supplementary fig. S7:
Original images of western blotting used for figure 4c (shown as cropped images in Figure 4).

Suppl fig. S8:

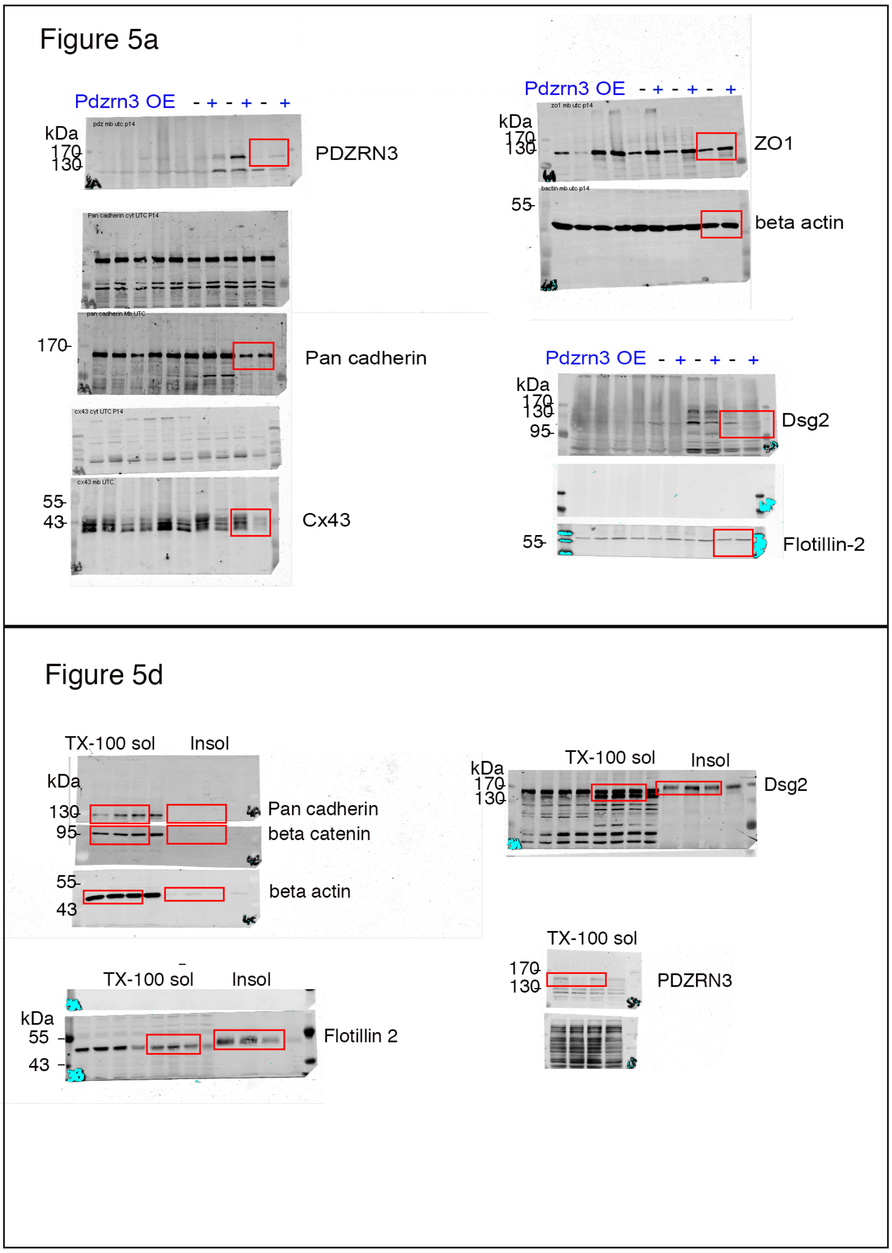

Supplementary fig. S8:
Original images of Western blotting used for figure 5a and 5d (shown as cropped images in Figure 5).

Suppl fig S9

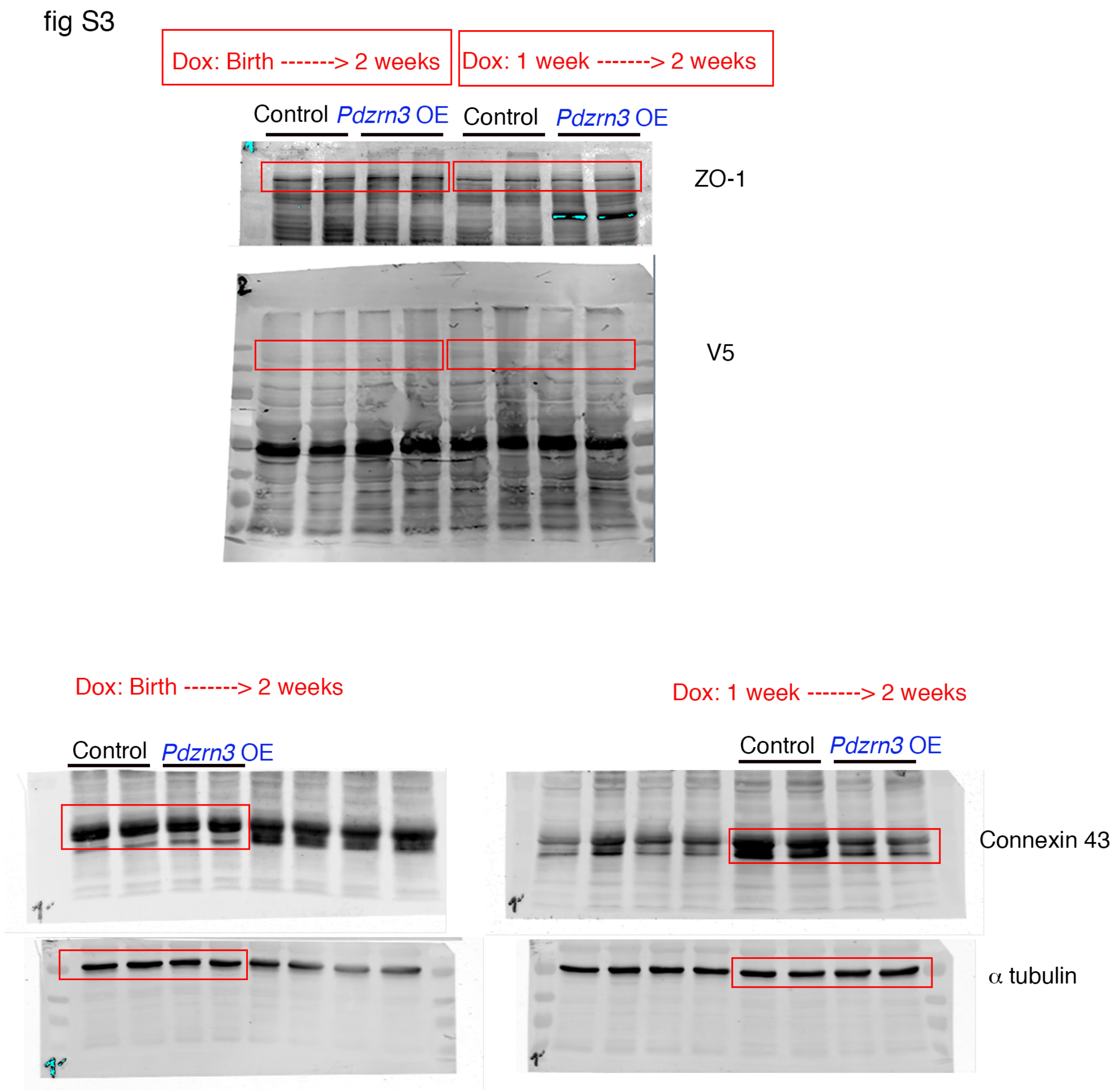

Supplementary fig S9: Original images of western blotting used for Supplementary figure S3 (shown as cropped images in Supp figure S3).

Suppl fig. S10:

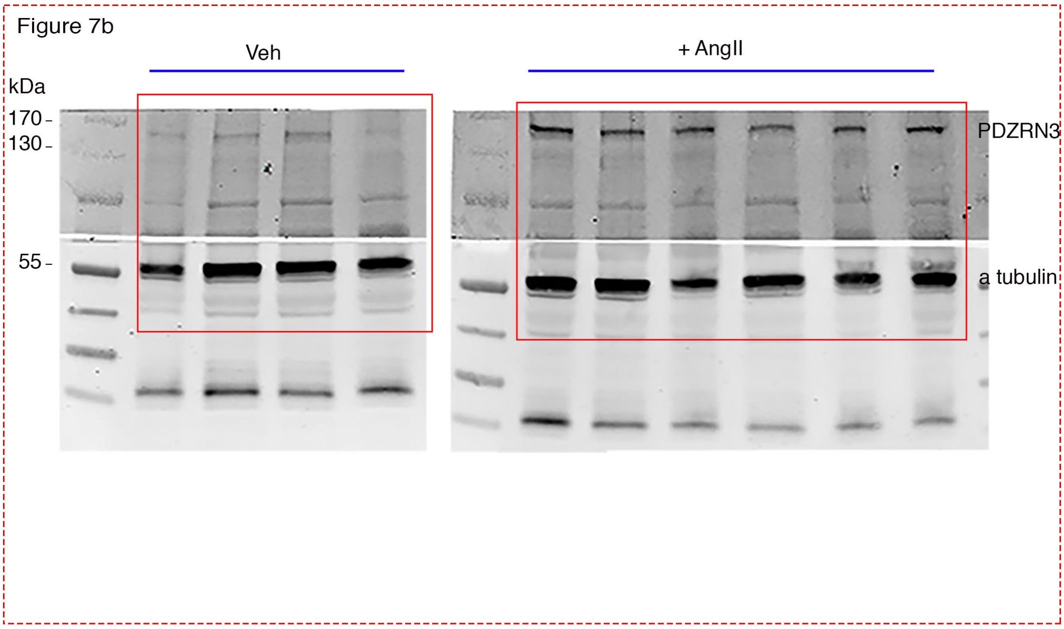

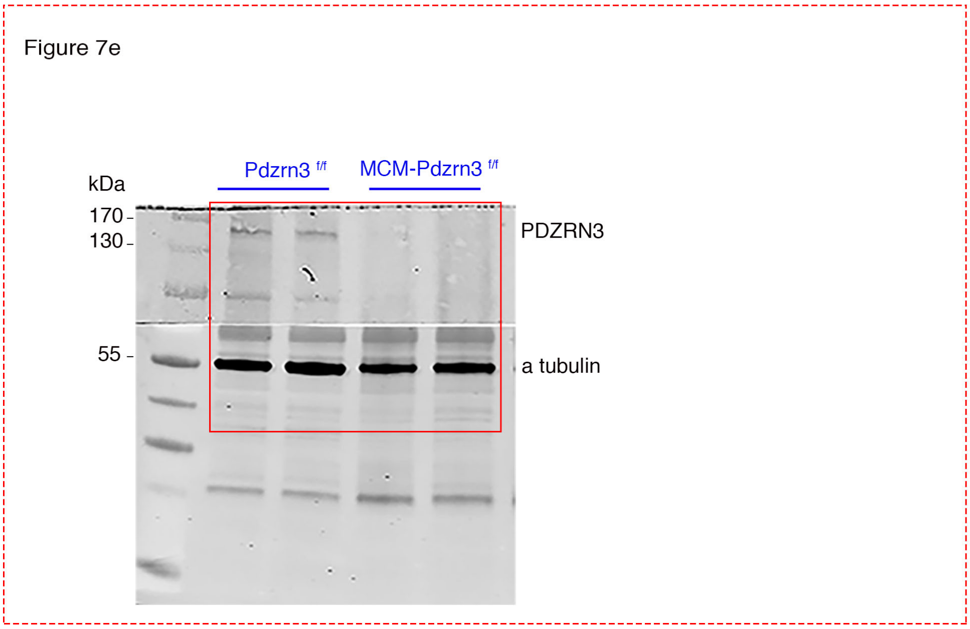

Supplementary fig. S10: Original images of Western blotting used for figure 7b and 7e (shown as cropped images in Figure 7).
